# A side-effect free method for identifying cancer drug targets

**DOI:** 10.1038/s41598-018-25042-2

**Published:** 2018-04-27

**Authors:** Md. Izhar Ashraf, Seng-Kai Ong, Shama Mujawar, Shrikant Pawar, Pallavi More, Somnath Paul, Chandrajit Lahiri

**Affiliations:** 1 The Institute of Mathematical Sciences, Chennai, 600113 India; 2 B.S. Abdur Rahman Crescent Institute of Science & Technology, Vandalur, Chennai, 600048 India; 3 Department of Biological Sciences, Sunway University, 47500 Petaling Jaya, Malaysia; 4 Department of Computer Science & Department of Biology, Georgia State University, Atlanta, GA 30303 USA; 5 Department of Bioinformatics, University of Pune, Pune, Maharashtra 411007 India; 6 Department of Computer Science and Engineering, Birla Institute of Technology, Mesra, India

## Abstract

Identifying effective drug targets, with little or no side effects, remains an ever challenging task. A potential pitfall of failing to uncover the correct drug targets, due to side effect of pleiotropic genes, might lead the potential drugs to be illicit and withdrawn. Simplifying disease complexity, for the investigation of the mechanistic aspects and identification of effective drug targets, have been done through several approaches of protein interactome analysis. Of these, centrality measures have always gained importance in identifying candidate drug targets. Here, we put forward an integrated method of analysing a complex network of cancer and depict the importance of **k**-core, **f**unctional connectivity and **c**entrality (KFC) for identifying effective drug targets. Essentially, we have extracted the proteins involved in the pathways leading to cancer from the pathway databases which enlist real experimental datasets. The interactions between these proteins were mapped to build an interactome. Integrative analyses of the interactome enabled us to unearth plausible reasons for drugs being rendered withdrawn, thereby giving future scope to pharmaceutical industries to potentially avoid them (e.g. ESR1, HDAC2, F2, PLG, PPARA, RXRA, etc). Based upon our KFC criteria, we have shortlisted ten proteins (GRB2, FYN, PIK3R1, CBL, JAK2, LCK, LYN, SYK, JAK1 and SOCS3) as effective candidates for drug development.

## Introduction

Cancer, one of the world leaders in morbidity and mortality, reportedly causing 8.8 million of human global death in 2015 (http://www.who.int/mediacentre/factsheets/fs297/en/), needs much weighted attention. This highly complex phenomenon encompasses a multi-step process of the conversion of benign cells into malignant, with each step corresponding to the breakdown of the normal cellular control mechanism. Such effects on cell growth and differentiation are manifested through genetic abnormalities attributed to either heritability or extraneous agents like chemical carcinogens, radiations and infectious organisms (https://ntp.niehs.nih.gov/pubhealth/roc/listings/index.html). At the cellular level, a multitude of signal transduction pathways are evoked encompassing the involvements of the above^1^. This essentially incorporates numerous genes and proteins along with their involved interactions. Systems biological studies, including but not limited to network analyses, have been done to simplify the complexity posed by the huge volume of transcriptomic and genomic data related to cancer^2–6^.

Identification of therapeutic drug targets has taken a new dimension with the advent of network analyses^7,8^. Till date, networks of interacting proteins in disease pathways have been conferred as interactomes and analysed for identifying candidate drug targets^7,9,10^. The analyses focussed on some selected centrality measures that reflect the importance of a particular protein connecting major hubs of interacting protein partners in the complex disease scenario. For instance, an interactome of cancer proteins with centrality measures has been reported where the proteins have stronger interactions than the non-cancer disease proteins and products of essential housekeeping genes^8,11–14^. However, despite such efficient measures, drugs designed for identified targets were reported to have serious side effects^15^ to the extent of attaining the withdrawn status^16^. In fact, BC and DC of drug targets had been associated with toxicity^13,15,16^. Here, we present an integrated stepwise approach encompassing separate parametric measures of identifying potential cancer drug targets with a minimal risk of being declared as withdrawn at a later stage.

## Results

### Interactome Construction from Pathways

**Figure 1:**
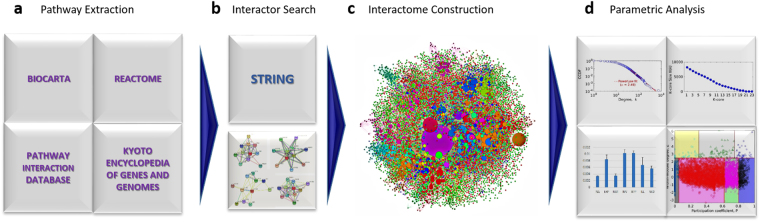
The approach for CaI construction and analysis. (**a**) The three databases BIOCARTA, PID, REACTOME and KEGG utilised for the extraction of pathways followed by disintegration into protein constituents and identification of any other pathways they are involved with. (**b**) The meta database STRING for finding the interactions of all the proteins pooled above. (**c**) The large component of the CaI constructed from pooled interactions above, coloured by 335 modules by Rosvall Algorithm with node size plotted as per degree (**d**) The analyses for power law, K-core, inter- and intra-modular connectivities for CaI constructed followed by the drug statuses against centrality measures, in clockwise manner.

We have built the complex interactome of proteins by integrating the interactions of protein components of all the pathways related to cancer (Fig. 1). These pathways of cancer are the established ones taken from four separate databases namely, BIOCARTA, Pathway Interaction Database (PID), REACTOME and Kyoto Encyclopedia for Genes and Genomes (KEGG) (Fig. 1a). The interactome is comprised of 129,276 interactions (as per STRING database) arising from 8,177 proteins contributing to the Large Connected Component (LCC) having 335 functional modules (Fig. 1b,c). The LCC is hereafter referred to as the Cancer Interactome (CaI) and analysed for obeying power law, determining core proteins and classifying proteins as per functional module connectivity, centrality measures, and drug status (Fig. 1d). All data pertaining to CaI analysis are in Supplementary Data 1.

### Network Topological Analyses

**Figure 2:**
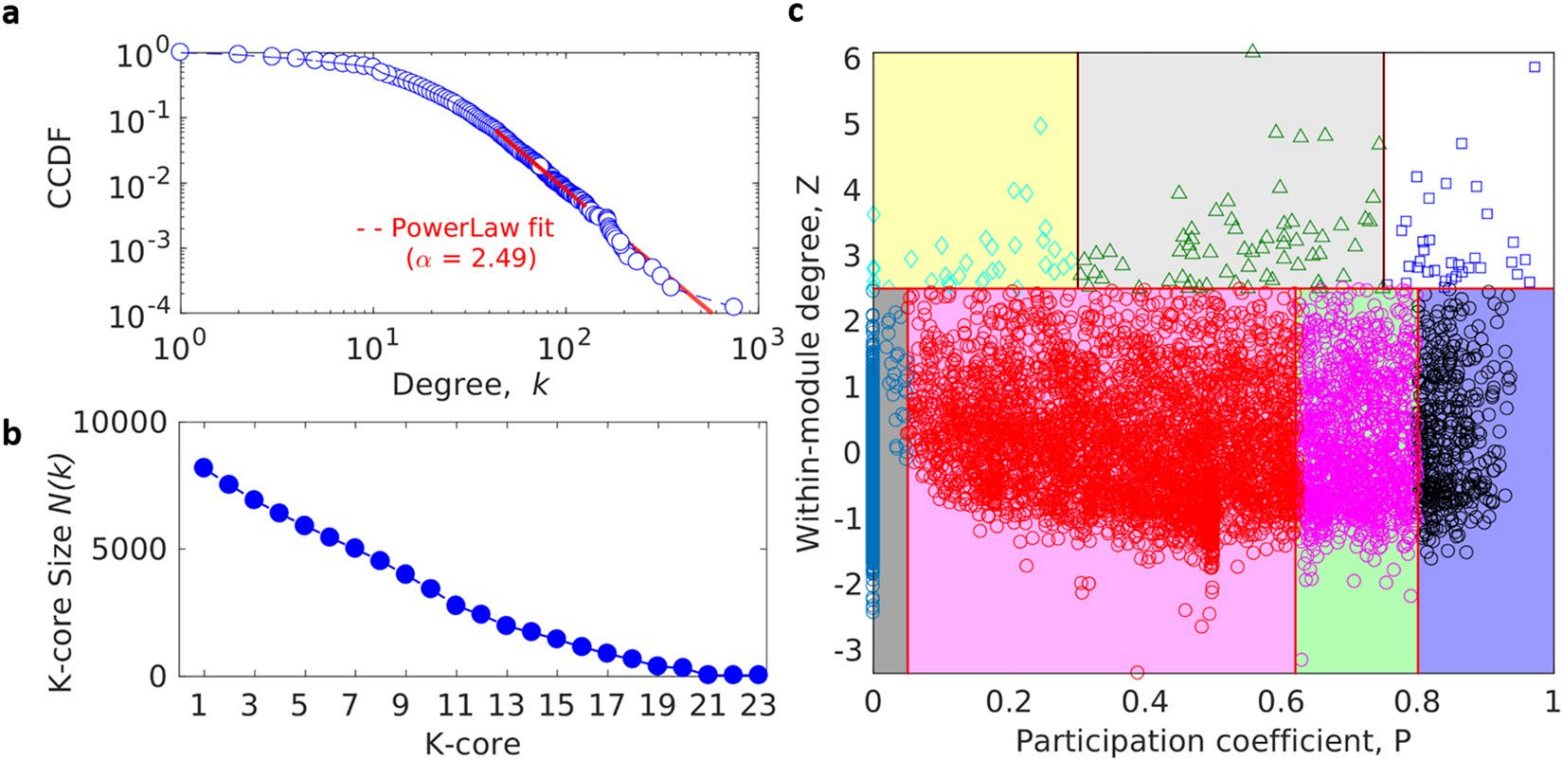
The technical analysis of the constructed CaI. (**a**) Complementary Cumulative Degree Distribution (CCDF) of CaI showing Power-Law behaviour. (**b**) K-core analysis of CaI representing the size of each k-shell (number of proteins appearing in k-core but not in k + 1th core) from periphery (k = 1) to inner core (k-max). (**c**) Classification of CaI proteins (R) based on its role and region in network space, the P-Z space classified into 7 categories of hub and non-hub nodes. The latter has been assigned as ultra-peripheral (R1), peripheral (R2), non-hub connector (R3) and non-hub kinless nodes (R4) and the former has been assigned as provincial (R5), connector (R6) and kinless hubs (R7) as described by Guimera *et al*.^17^. Kinless hubs nodes are supposed to be important in term of functionality, which has high connection within module as well as between modules.

Our CaI obeys power law and shows a merger of the blue dots from the empirical data with the red dotted lines thereby suggesting scale free nature of the network (α = 2.49) (Fig. 2a). A degree preserved randomized network yielded similar scale free nature with α = 2.49 (Supplementary Fig. 1a). The k-core analysis of the CaI categorized it into 23 shells (Fig. 2b) compared to 13 shells for the random network (Supplementary Fig. 1b). Thus, a further decomposition of the CaI disintegrates the network completely which makes 23^rd^ as the innermost shell. While this conveys the importance of the proteins belonging to the 23^rd^ shell, a further classification of the proteins based on the inter- and intra-modular connectivity was observed and reflected by the cartographic analysis of participation coefficient, P vs. z-score (Fig. 2c). This approach classified the CaI proteins into two major categories of non-hub (low z; z < 2.5) and hub nodes (high z; z ≥ 2.5). In a network scenario, the non-hubs can be either ultra-peripheral (R1; low z and ultra-low P), peripheral (R2; low z and low P), connector (R3; low z and moderate P) and kinless (R4; low z and high P) whereas above a certain threshold, all other hubs were classified as provincial (R5; high z and low P), connector (R6; high z and moderate P) and kinless (R7; high z and high P). Notably, for a degree preserved randomized network the cartographic representation of the proteins showed limited inter- and intra-modular connectivities.

### Drug Status of Proteins in Functional Modules

**Figure 3:**
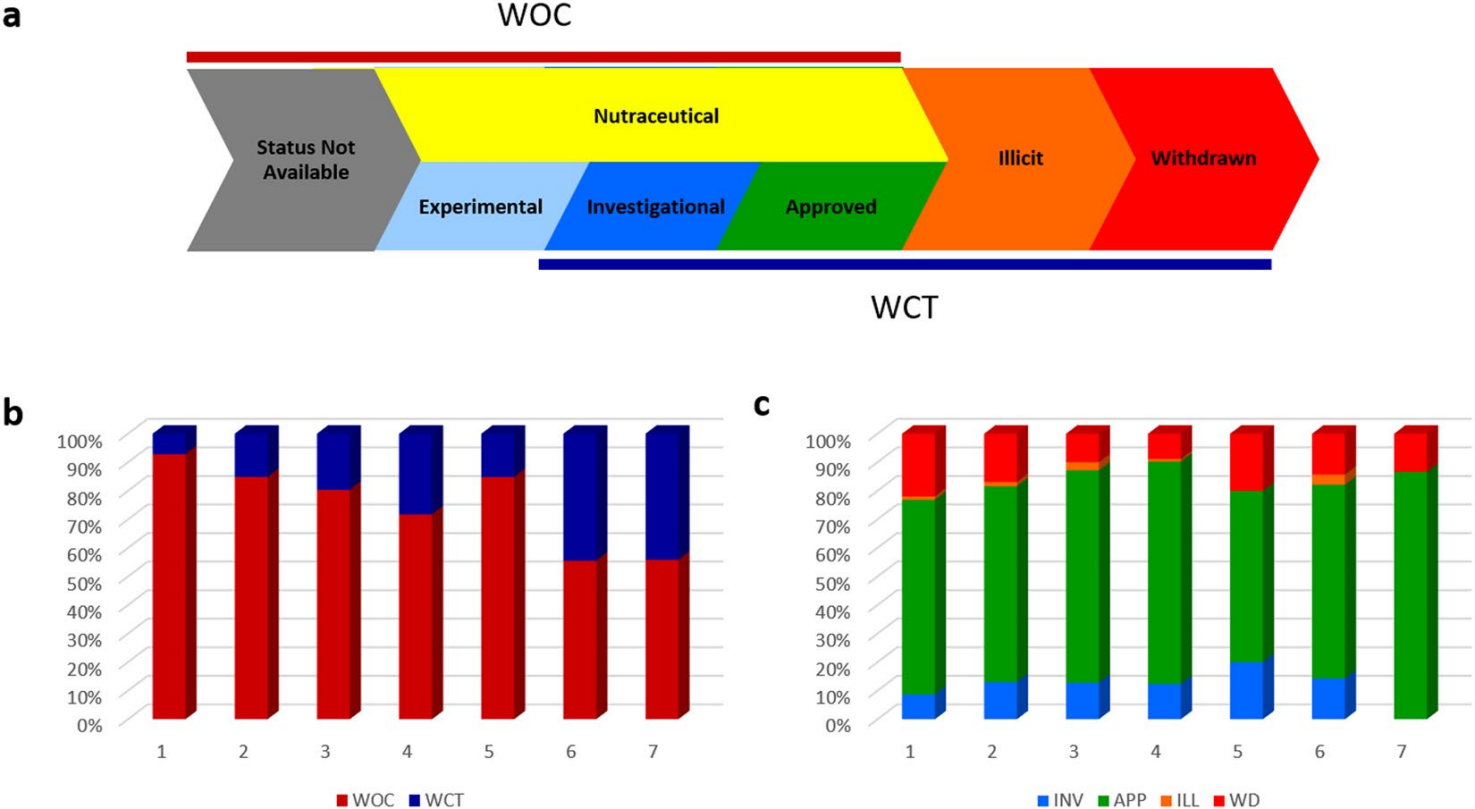
The drug statuses of the CaI proteins. (**a**) The hierarchical statuses of the drugs based on the information from Drugbank database and FDA regulations. Nutraceuticals (shaded in yellow) need not undergo clinical trial (WCT) and projects out from the experimental phase (WOC). (**b**) Segregation of classified R proteins having WCT (blue bars) and WOC phases (in red). R1: n = 2422; R2: n = 3953; R3: n = 1272; R4: n = 400; R5: n = 33; R6: n = 63; R7: n = 34. (**c**) Classification of R proteins (Y-axis) into the drug status of investigational (INV, light blue) or higher viz. APP (green), ILL (orange) and WD (red). R1: n = 173; R2: n = 598; R3: n = 250; R4: n = 113; R5: n = 5; R6: n = 28; R7: n = 15.

We have hierarchically set the status of the drugs which are available for targeting the proteins of our CaI (see methods section). While proteins of the class nutraceutical (NUT) and experimental (EXP) belong to drugs without clinical trials (WOC), those falling under investigational (INV), approved (APP), illicit (ILL) and withdrawn (WD) statuses have, at least, undergone the clinical trials (WCT) (Fig. 3a). All our target proteins from CaI were initially classified into either WOC or WCT categories (Fig. 3b) across the seven different functional modules (R-groups). A gradual increment of the percentage of proteins having WCT statuses was observed across R1 till R7, with R5 as an exception. Conversely, a gradual decrease was observed for the proteins having WOC statuses with a similar exception of R5 group. Both WOC and WCT categories of proteins had similar percentage of drugs in R6 and R7. An in-depth picture of the target proteins from WCT was obtained from a segregation into INV, APP, ILL and WD statuses across R1-R7 groups (Fig. 3c). We observed a huge proportion of approved drug statuses throughout all the functional categories of proteins. Moreover, R5 and R7 did not show any ILL status. Additionally, R1 has the highest proportion of WD status followed by R5, R2 and R7. Notably, R5 has the highest proportion of INV status for the targets while R7 did not show any.

### Effective drug statuses of proteins with high EC values

**Figure 4:**
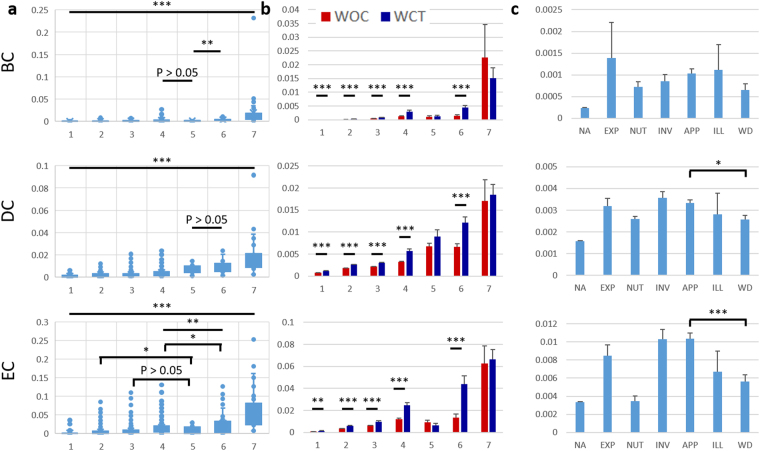
Network centrality impact of the classified CaI proteins upon drug statuses. (**a**) The distribution of the centrality measures of betweenness (BC), degree (DC) and Eigenvector (EC) for the classified R proteins in the x-axis: R1, n = 2422; R2, n = 3953; R3, n = 1272; R4, n = 400; R5, n = 33; R6, n = 63; and R7, n = 34. The mean is shown through the box and whisker plot. (**b**) The mean distribution of the BC, DC and EC measures for the classified R proteins having WOC and WCT statuses for the drugs developed against them in the x-axis. For WOC: R1, n = 2249; R2, n = 3355; R3, n = 1022; R4, n = 287; R5, n = 28; R6, n = 35; and R7, n = 19 and for WCT: R1, n = 173; R2, n = 598; R3, n = 250; R4, n = 113; R5, n = 5; R6, n = 28; and R7, n = 15. (**c**) Impact of centrality measures on the individual drug statuses of the classified R group proteins. X-axis represents different drug statuses: Not Available (N/A; n = 6193), Experimental (Exp; n = 287), Nutraceutical (NUT; n = 515), Investigational (Inv; n = 144), Approved (APP; n = 837), Illicit (ILL; n = 20), and Withdrawn (WD; n = 181). Only the statistical comparison between APP and WD are shown in the graph (refer to Supplementary Table 2 for more in-depth statistical results). Y-axis of the graphs represents centrality measures BC, DC and EC for respective panels in rows. Error bars = standard error, *p < 0.05, **p < 0.01, ***p < 0.001.

As centrality measures of betweenness (BC), degree (DC), and eigenvector (EC) have generally been used for uncovering the essential nature of a particular protein, we have observed the distribution of these measures for our CaI proteins across the different R-groups (Fig. 4a). While the average centrality measures for the groups from R1 to R7 are ascending for BC and DC, EC gives a different reflection (Fig. 4a). Notably, the average centrality values for the proteins of R4 and R5 categories are significantly different for both DC and EC but not for BC. Again, centralities for proteins of R5 and R6 are significantly different for BC and EC but not for DC. Thus, R4, R6 and R7 are the groups of proteins with the top three highest average centrality values reflected by EC only.

On moving further for the proteins as targets undergoing clinical trials (WCT) compared to those without (WOC), significant differences were observed across all categories of proteins ranging from R1 till R6, with the exception of R5 or R7 (Fig. 4b). Notably, none of the centralities stands out in this comparison. On the contrary, a clearer comparison can be obtained when we compare the three types of average centrality measures across the different drug statuses (Fig. 4c). Interestingly, EC (p < 0.001) is shown to be a better measure to distinguish between APP and WD than BC (p > 0.05) and DC (p < 0.05) (Fig. 4c). A different reflection for identifying proteins with acceptable drug statuses was obtained from a comparison of the EC, DC and BC measures against the functional R-groups (Supplementary Fig. 2). As reflected in Fig. 4a, R7 has the highest values across all centrality measures. However, high centrality value cannot help to distinguish withdrawn status from the others in either of the centrality measures. Surprisingly, EC for R6 still stands out to be the clear indicator for identifying effective drug targets from CaI proteins (Supplementary Fig. 2).

### Integrative approach by k-core, functional R-group and centrality

Next, we develop a novel approach to identify drug target by integrating all results obtained from this study. We use **k**-core, **f**unctional connectivity and **c**entrality (KFC) as the key criteria for selecting the most effective drug targets expected to have the least chance for being rendered illicit and withdrawn. Based on the above criteria, we choose k-20 (20^th^ k-shell), function R-6 and eigenvector centrality value >0.03, for finding the ideal candidate proteins for drug targets. The top ten list of proteins generated by this approach are GRB2, FYN, PIK3R1, CBL, JAK2, LCK, LYN, SYK, JAK1 and SOCS3 (Table 1). Strikingly, this list is quite different from the lists generated by the conventional approaches of using betweenness, degree and/or eigenvector centralities (Table 1).

**Table 1:** The top rank proteins of CaI through conventional and KFC methods.

Sr. No.	Conventional Approach			Our Approach
	BC	DC	EC	k-20/R6/EC
1	UBC	UBC	UBC	GRB2
2	TP53	TP53	SRC	FYN
3	INS	SRC	AKT1	PIK3R1
4	AKT1	AKT1	TP53	CBL
5	SRC	INS	GRB2	JAK2
6	CALM1	FYN	EGFR	LCK
7	JUN	KNG1	STAT3	LYN
8	ALB	GRB2	FYN	SYK
9	EGFR	JUN	JUN	JAK1
10	DIF	ADCY2	PTPN11	SOCS3

## Discussion

Our CaI integrated the interactions of the proteins involved in the established pathways of cancer. It is to be noted that each node of CaI represents one gene product, protein or drug target. To address the pleiotropism of certain gene products, we have considered other cellular and signal transduction pathways encompassing the aforementioned proteins. Thus, each node of CaI has connections, interactions or links with the others that can be from either the same or different signalling pathway. Our way of building the CaI contributed to the highly complex and multi-factorial phenomenon of cancer in the real scenario. This was shown by the degree distribution of CaI with a power law behaviour having α exponent 2.49, which suggests the scale-free nature^18^ (Fig. 2a). Moreover, an idea of the complex interaction of the CaI proteins were obtained from the k-core analysis (Fig. 2b). Interestingly, the innermost (23^rd^) shell had the ribosomal proteins reported to be either directly related to cancer or involved in genetic and metabolic diseases having increased risks of malignancy^12^. This is followed by the 21^st^ shell wherein we have the ribosomal proteins once again. Notably, there are no proteins appearing in the 22nd shell, perhaps due to the absence of the proteins having 22 interactions. Thereafter, the 20^th^ shell had a barrage of the proteins reported to be directly involved in cancer phenomenon. These are AKT1, TP53, STAT3, CTNNB1, CDC42, CDK2, BRCA1, MAPK1, MAPK3 and CALM1 among others. With such a huge list of proteins involved in the complex phenomenon, it was essential to understand their functional relatedness, if any.

For viewing the functional connectivity of the proteins, we categorized them into seven classes (R1-7) with a ‘cartographic representation’ of the intra- and inter-modular connections, measured by z-score and participation coefficient, P, respectively, as proposed for analysing large complex networks^17^ (Fig. 2c). Notably, a module refers to a cluster of nodes interacting with each and does not always equal to a particular pathway whereas a hub refers to a node that has the most interactions with others within and between modules. Thus, each functional group of cartography represents group of nodes that share similar interaction profile, in terms of inter- and intra-modular interaction. Ten such random examples of R7 kinless (represented by high inter-modular connections) hubs (high intra-modular connections) are UBC, SRC, AKT1, TP53, PTEN, STAT3, ESR1, MAPK1, INS, and CTNNB1. This reflects that each of these proteins has roles in multiple signalling pathways (inter) as well as main roles interacting with other proteins within the same signalling pathway (intra). Hence, the drug target identified by our analysis is based on its relative importance in the CaI rather than the interactome of any single signalling pathway.

It is imperative that R7 proteins, with high intra- and inter-modular connectivity, are supposed to be important in terms of functionality. On this note, non-hub R1 and R2 proteins are least connected and can be pruned easily without much affecting the whole network^19^. Besides, non-hub R3 proteins are expected to take part in a few but fundamental set of interactions contrary to hub R5 proteins having many within-module connections. We observed similar homogeneous distribution between all modules of R4 and R7 (high P). Hence, those proteins having moderate (R6) to high (R4 and R7) P values are most conserved in terms of retaining these connections as essential ones for a particular system like CaI. All such essentialities emanating from R4, R6 and R7 proteins could be very crucial in considering them as potential drug targets. Efforts to find better measure for drug targets, with different approach, had been reported earlier, *albeit*, in a completely different scenario^20^.

To check out the robustness of our approach of CaI construction, we performed degree preserving randomization by comparing our empirical network with a network of randomized protein-protein interactions having preserved total interactors and degree (Supplementary Figure 1). The degree distribution of nodes, showing the power law behavior with the same exponent (α) of 2.49, are intact even after the randomization (Supplementary Fig. 1a). This is because the degree of nodes are preserved during the course of randomization. The comparative effect of randomization was clearly delineated through Supplementary Fig. 1b and c, of which the former shows the core-periphery structure of the randomized network with less number of cores compared to the empirical network. This is because randomization has interfered with the inherent structure. The latter figure shows the structural division of network-nodes based on the participation coefficient and within module Z-score. Due to randomization, all the nodes got accumulated only in fewer sections (R1, R2 and R5), thereby reflecting the absence of a specific structure in the network. This approach preserved the overall structure of the network and ensured that the results are not an artefact of the inherent structural properties of network. The randomization process strengthened our empirical study by confirming that the distribution of nodes in the section R6 and R7 of empirical case is not by random chance.

Conventional methods of selecting protein targets for drug development have the potential risk of the drugs being later banned for therapeutic use in human. Therefore, to evaluate the sustainability of the protein targets, we have used 5477 drugs from Drugbank and hierarchically set the different statuses of INV, APP, ILL and WD from those under WCT categories^21–23^ (Fig. 3a). Among these, drugs with ILL and WD can confer either toxicity or ineffectiveness and thus, demand a more effective approach towards identification, selection and approval during the process of drug development.

To understand the effect of functional connectivity of protein targets upon the drugs being developed, we analysed whether the distribution of the drug status differs according to the different functional groups (R1 to R7) of cartographic representation. Our CaI has higher percentage of proteins from the R4, R6 and R7 categories falling into the WCT group (Fig. 3b). On the contrary, R1 and R5 have higher WD surpassing R6, R7 and R4 (Fig. 3c). Again, while neither R5 nor R7 reflects the ILL status, R6 has the highest (Fig. 3c). Moreover, R5 has the highest proportion of INV proteins compared to other functional R groups. Thus, despite giving a faint idea, a clear direction for an effective selection of the drug target still remains elusive.

To gain more insights for a directional approach of drug target selection, we have resorted to the centrality measures like BC, DC and EC which have been either targeted for drugs, as mentioned earlier, or have been in focus for being important and indispensable for gene-disease associations^14,16,23,24^. For a total of 8177 proteins analysed, we found that EC has higher average distributions for R4, R6 and R7 categories of proteins compared to BC and DC. R7 group of proteins has the highest values throughout all the three centrality measures which implies their importance as drug targets. Despite such importance of R7 group of proteins, R4 and R6 also gained prominence through EC measures only (Fig. 4a). The top ten proteins with high EC measures from the different functional R-groups are UBC (R7), SRC (R7), AKT1 (R7), TP53 (R7), GRB2 (R6), EGFR (R4), STAT3 (R7), FYN (R6), JUN (R4) and PTPN11 (R3) (Table 1). Except the last one, all of these are mixtures of R7, R4 and R6 as can be reflected from the parentheses. These findings are coherent with our list of genes being grouped under the highlighted EC measures with those from an attempt by Wang *et al*. to find the important genes for breast cancer^14^.

To assess the predictability of the centralities for drug target potential, we divided the total proteins into WOC and WCT targets and calculated their average centrality measures for each functional groups. Strikingly, R7 and R5 categories of proteins did not show significant differences of centrality values between WOC and WCT groups which are shown by R4 and R6 proteins (Fig. 4b). Such significant differences suggest there are more focus on the proteins from WCT status than those of WOC categories. Again, for those which did not show the difference, have similar proportion of the proteins under WOC and WCT statuses within each functional R category. Thus, for WOC and WCT proteins, which shared almost similar centrality profiles, we hypothesize that some proteins under the former group could be achieving similar drug status for drug development as the latter group.

Next, to determine whether hierarchical drug statuses can be distinguished by centralities, we mapped the former against the average values of the latter. Our finding shows that values of APP, ILL and WD drug statuses from either BC or DC are not clearly distinguishable in giving the guidance towards target selection. On the contrary, EC is a better indicator than BC and DC for distinguishing potential APP (average EC = 0.010341) from ILL (0.006735) and WD (0.005624) targets (Fig. 4c). However, the caveat is that the centrality values rely on the assumption of all targets having the same functional connectivity. Thus, we postulated that targets with different functional connectivity (in this case, R groups) must have different set of centrality values to distinguish APP from ILL and WD.

To unravel the different drug statuses within the same R groups, we categorised the targets in them with the average centralities to gain a better picture for the target selection. Interestingly, we observed that neither of the centrality measures clearly distinguish between the APP and WD statuses in R7 (Supplementary Fig. 2). This means that the highest centralities are given to proteins which get mostly targeted for the drugs attaining either the APP or WD statuses at a later point of time. This is expected as proteins targeted in R7 are the most important in terms of their inter- and intra-class interactions. However, the reason of the ILL status completely missing from the R7 categories, remains elusive.

Again, ILL statuses with high BC and DC measures in R6, makes them unfit for target selection. Notably, ILL status is also not observed for R5 across all centralities. The centralities values of the statuses are nominal in R1-3 making the proteins less impactful as targets. This could be possible because either these proteins are not biologically relevant or they were not extensively worked upon before. The statuses in R4 can still be considered for a target selection, but their overall values are relatively low. In contrast, EC shows R6 to be the most useful category for drug target selection as it can clearly distinguish between the targets with APP status (EC = 0.048708) and those with either ILL or WD statuses (0.02746801 or 0.026112, respectively) (Supplementary Fig. 2). This finding implies that we can utilize the R6 targets with high EC values (>0.03) as the threshold to avoid potential risk of being illicit and withdrawn. Furthermore, we found that the proteins in k-20 shell is more suitable for potential drug target compared to k-23 and k-21 which comprise ribosomal proteins. Even though the importance of ribosomal proteins as drug targets is in practice lately^25^, the risk factor and the toxicity issue should also be considered as reported by Bottger *et al*.^26^. Moreover, the ribosomal proteins, incorporated within the k-23 and k-21 shells of our analytical study, belong to the R1, R2 and R5 functional groups which were never in focus with respect to our drug targets. Thus, we conclude that, for a general safeguard practice, the combination of k-20, R6 groups along with eigenvector centrality measures with a proper threshold is the recommended approach to identify effective drug targets (Table 1). In fact, PIK3R1 from our list serves to be the regulatory subunit 1 of the enzyme Phosphoinositide-3-Kinase, the catalytic subunit of which has been reported to be important for breast cancer along with a k-15, R6 list holder, Fms-Like Tyrosine Kinase (FLT1) gene^27^.

Finally, our approach as delineated above, gained relevance from a mapping of the different biological aspects of the CaI proteins related to molecular function (MF), biological process (BP), cellular component (CC), protein class (PC) and pathway (PW), against the seven R categories with WCT statuses (Supplementary Figs 3–7). We do not find any distinct difference between the proteins in terms of MF, BP, CC and PC. However, the PW pie chart for these proteins clearly reveals the pleiotropicity of the genes due to the cross-talk among signaling pathways (Supplementary Fig. 7). A closer look at the gene ontology (GO) of the R1-7 proteins having different categories of drug statuses revealed the uniqueness of each group, providing insight about the unique factor that contributes to the likelihood of a particular drug status (Supplementary Figs 8–19). For instance, drugs that target enzyme modulator (R4, R7), macromolecular complex (R5, R6), transferase (R5, R6, R7), membrane protein (R6), and hydrolase (R7) are potential candidates to be approved. On the contrary, drugs meant to target cytoskeletal proteins (R4, R6, R7), transcription factors (R4, R6), receptor proteins (R4, R7) are likely to be withdrawn (Supplementary Figs 14–19).

Our integrative KFC method is a guideline for identification of the potential candidates for investigation and carrying forward for approval during the drug developmental process. A close following of such method would lead to drugs being developed with little or no risk of obtaining illicit or withdrawn statuses. Ideally, our CaI analysis identified those are the ones which belongs to k-20^th^ shell, shown to have moderate inter- and high intra-module connectivity (R6) carrying high eigenvector centrality (>0.03 for R6).

## Methods

### Dataset Collection

Data for constructing the Cancer Interactome (CaI) were collected in two steps. Initial ones were obtained by disintegrating the component proteins of the established pathways of cancer from four separate databases namely, BIOCARTA, Pathway Interaction Database (PID), REACTOME and Kyoto Encyclopedia of Genes and Genomes (KEGG)^28–30^. To address the pleiotropic effect of the gene products, the aforementioned proteins were then used to search for their involvement in other cellular and signal transduction pathways related to cancer listed in the same databases. Pathways of the second categories were disintegrated to their protein components and were added to the initial pool of proteins mentioned above. Thus, we pooled the individual interacting proteins from all such aforesaid pathways after removing the redundant interactions arising from the involvement of the same proteins in different pathways. UniProt IDs were used along with gene name identifiers for pooling a total set of 8,177 unique proteins (last date of access: January 2018).

### Interactome Construction

The accession IDs mentioned above were fed as queries to the STRING version 10.0 biological meta-database^31^ to obtain 129,276 interaction information of 8,177 proteins. Essentially, the interaction information of all the proteins, weighted by the combined scores from different parameters as per STRING, was imported into Gephi version 0.9.1^32^ for visualization. The interactome of cancer proteins, thus constructed, can be perceived as the protein interaction network (PIN) represented by an undirected graph G = (V, E) consisting of a finite set of V vertices (or nodes) and E edges^33^.

### Network Analyses

Our CaI was analysed by using MATLAB version 7.11, a programming language developed by MathWorks^34^. For the primary understanding of the network, we have plotted distributions of network degree (k) by Complementary Cumulative Distribution Function (CCDF), which follows the power law distribution. This is stated by the probability of nodes having degree at least k such that F(K) = P(K ≥ k).

To have an idea of the cohesiveness of each protein of CaI, we performed the k-core analysis, which is a recursive pruning technique of network^19^. A k-core is a subnetwork in which all nodes have at least k-connection. All nodes, which are in k-core but not in (k + 1)-core is referred as the k-shell. This is able to classify the nodes (proteins/genes in our study) based on the variety of their interacting partners. Protein/gene which belongs to outer shell (lower k value) is having limited number of interacting partner protein and are very specific, while those belonging to inner k-core/shell are very common proteins, which might have variety of roles to play.

For further analysis, we have classified the proteins of CaI based on their role and region in network space, which is defined by the within module Z-score of each node and its participation coefficient, P, respectively^17^. Based on the network ‘module’ concept of Rosval^35^, the proteins are conceived to be having a measure of their intramodular connectivity with other proteins, namely z-score. Furthermore, a participation coefficient, P, of the proteins measures their inter-modular relatedness. A degree preserving randomization of the CaI was performed with total number of degree and interactors being kept intact.

The CaI was subsequently analysed to compute values for the network centrality parameters namely degree centrality (DC) and betweenness centrality (BC) and eigenvector centrality (EC). Degree centrality (k) of a protein in protein interaction network is defined as number of direct interaction with other kind of protein, which can be interpreted as the versatility or the capability of interacting with other kind of proteins/genes) of a protein in the system. Highly versatile protein might consider as an important factor in many cases. The betweenness centrality of a node is defined as the fraction of shortest paths between pairs of vertices in a network that pass through the node of interest *i*. Eigenvector centrality is characterized by the ‘global’ importance of a node in a network, which is calculated from eigenvector of network matrix. A node is considered to be higher EC, if it is connected with high degree nodes.

### Setting Drug Hierarchy

We have hierarchically set the different statuses of **5477** drugs as mentioned conventionally in Drugbank^22^. Essentially, drugs are classified as either dealt with clinical trials (WCT) and those without such trials (WOC). The former comprises proteins attaining the ‘investigational’ status and above in the hierarchy with at least clinical trials initiated. The latter has the targets with no known drug(s), experimental or nutraceutical status. Biomolecules like ATP and amino acids acting as supplements of standardized nutrients are termed as nutraceuticals (NUT) which are considered as drugs of some pharmaceutical grades. Ideally, a chemical compound that has been proven experimentally to bind specific protein targets is considered as a drug. Upon passing the experimental phases, the drug entering the different phases of clinical trials is categorised as investigational (INV). Upon completion of clinical trials, the drug achieves the status of being approved (APP)^21^. Despite such approval for human consumption, some drugs start giving harmful side effects in some population which are designated as illicit (ILL). Drugs withdrawn from the market and banned for further therapeutic usages are given WD status^23^. We examined the drugbank for the total number of drug statuses per protein target and then considered the highest status that each protein target (a.k.a drug target) can attain.

### Statistical Significance Tests

We have used the Kruskal-Wallis H test (or Mann Whitney U test when warranted by IBM SPSS) as the rank-based non-parametric test to determine if there are statistically significant differences between two or more groups of independent variables like functional modules and drug statuses on a continuous or ordinal dependent variables like centrality measures BC, DC and EC. All such statistical tests were performed using IBM SPSS version 21 (IBM Corp. Released 2012. IBM SPSS Statistics for Windows, Version 21.0. Armonk, NY: IBM Corp.).

## Electronic supplementary material

Supplementary Information

Supplementary Data 1

Supplementary Data Statistics
