# Supplementary material for: A side-effect free method for identifying cancer drug targets
Source: Sci Rep. 2018 Apr 27;8:6669. doi: 10.1038/s41598-018-25042-2 (PMC5923273; doi:10.1038/s41598-018-25042-2)
Supplement: Supplementary file 1 — Supplementary Information [file 41598_2018_25042_MOESM1_ESM.docx]

**A side-effect free method for identifying cancer drug targets**

**Authors:** Md. Izhar Ashraf **^1, 2^** ^#^, Seng-Kai Ong**^3^** ^#^, Shama Mujawar**^3^**, Shrikant Pawar**^4^**, Pallavi More**^5^**, Somnath Paul**^6^** and Chandrajit Lahiri**^1, 3^** *

**Addresses: ^1^** The Institute of Mathematical Sciences, Chennai - 600113, India

**^2^** B.S. Abdur Rahman University, Vandalur, Chennai - 600048, India

**^3^** Department of Biological Sciences, Sunway University, 47500 Petaling Jaya, Malaysia

**^4^** Department of Computer Science & Department of Biology, Georgia State University, Atlanta, GA 30303, USA

**^5^** Department of Bioinformatics, University of Pune, Pune, Maharashtra - 411007, India

**^6^** Department of Computer Science and Engineering, Birla Institute of Technology, Mesra, India

# These authors have contributed equally to the work

**Correspondence:** *Chandrajit Lahiri, **E-mail:** chandrajitl@sunway.edu.my

**Phone: +60 3-7491 8622 Extn. 7174   Fax: +6 03 5638 7177**

**Authors :** Md. Izhar Ashraf, E-mail : [ashraf@imsc.res.in](mailto:ashraf@imsc.res.in)

Ong Seng-Kai, E-mail : [ongsk@sunway.edu.my](mailto:ongsk@sunway.edu.my)

Shama Mujawar, E-mail : [shama.m@imail.sunway.edu.my](mailto:shama.m@imail.sunway.edu.my)

Shrikant Pawar, E-mail : [spawar2@student.gsu.edu](mailto:spawar2@student.gsu.edu)

Pallavi More, E-mail : [pallavimore02@gmail.com](mailto:pallavimore02@gmail.com)

Somnath Paul, E-mail: [somnathpal49@gmail.com](mailto:somnathpal49@gmail.com)

Chandrajit Lahiri, E-mail : [chandrajitl@sunway.edu.my](mailto:chandrajitl@sunway.edu.my)


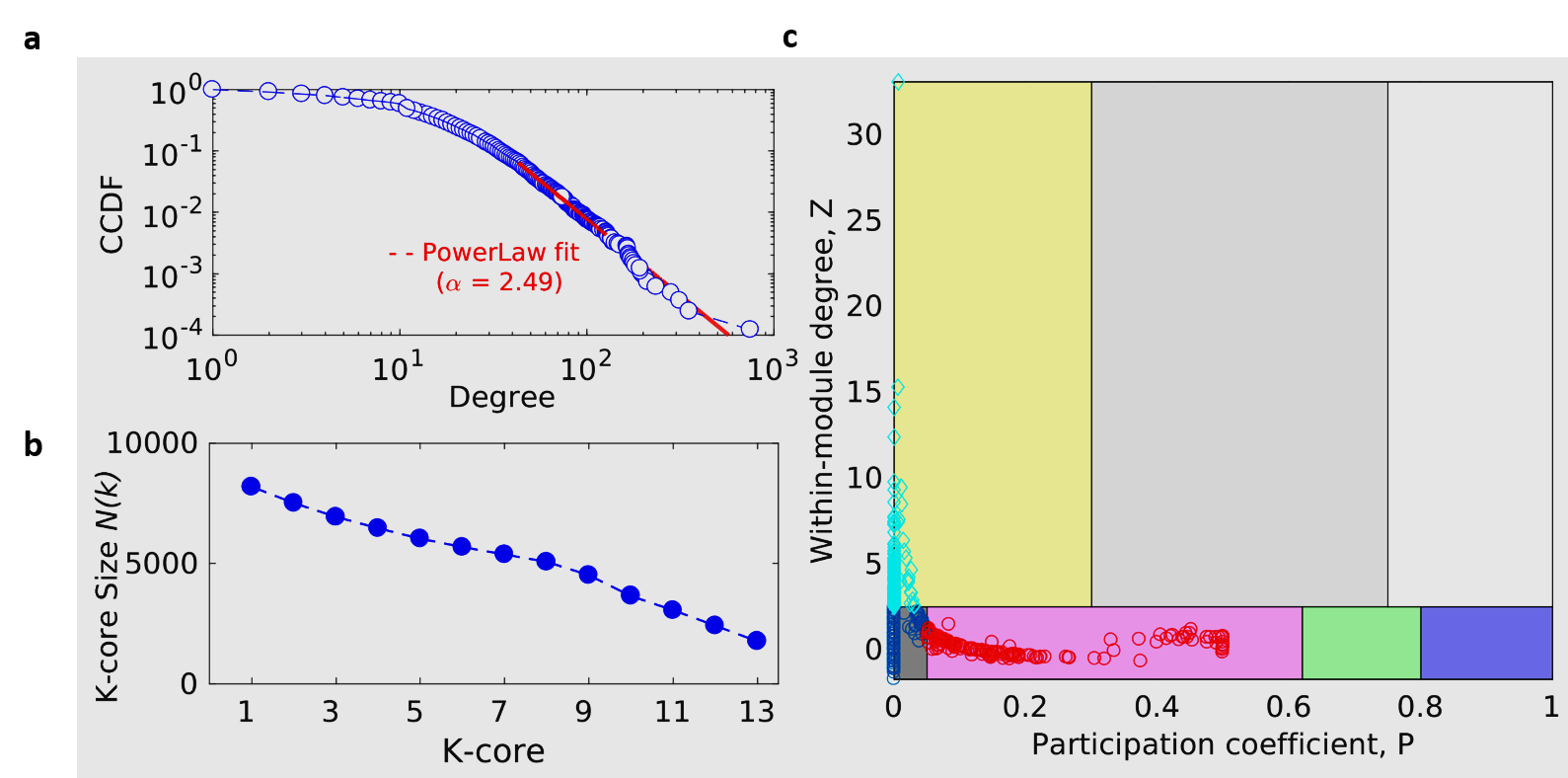

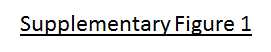


**Supplementary Figure 1. The degree preserved randomization of constructed CaI (a)** Complementary Cumulative Degree Distribution (CCDF) of randomized CaI showing same Power-Law behaviour. **(b)** K-core analysis of randomized CaI showing lesser numbers of k-shell. **(c)** Classification of randomized CaI proteins (R) based on its role and region in network space, the P-Z space classified into 7 categories of hub and non-hub nodes. The proteins got clustered in the ultra-peripheral (R1), peripheral (R2) and the provincial (R5) zones only.


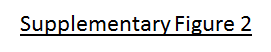


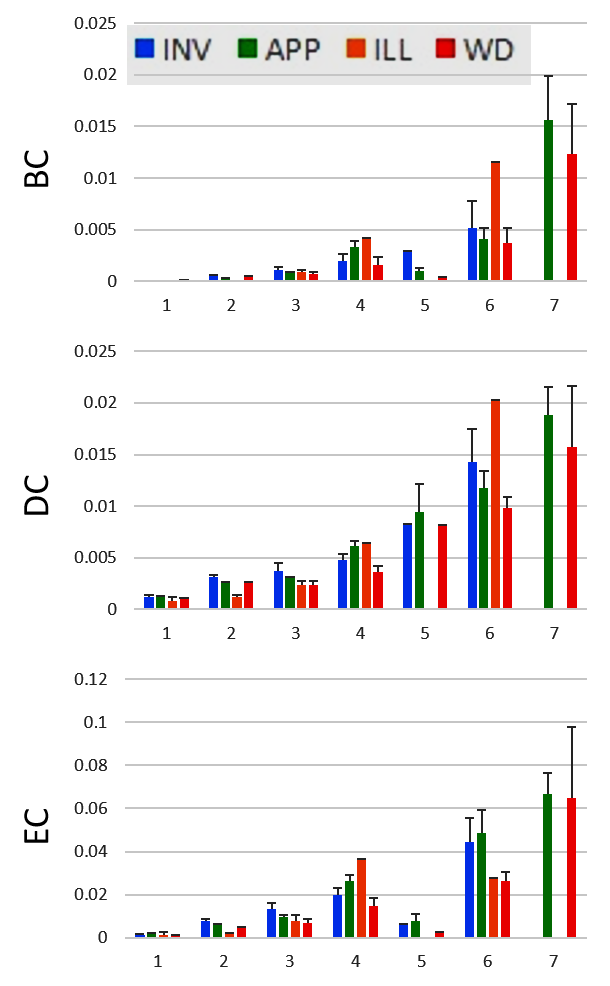


**Supplementary Figure 2. Impact of centralities on the different drug statuses within the same R groups.** The bar graphs show the comparison for the R1 to R7 groups of proteins of CaI. All the panelled figures run from top to bottom for BC, DC and EC measures.


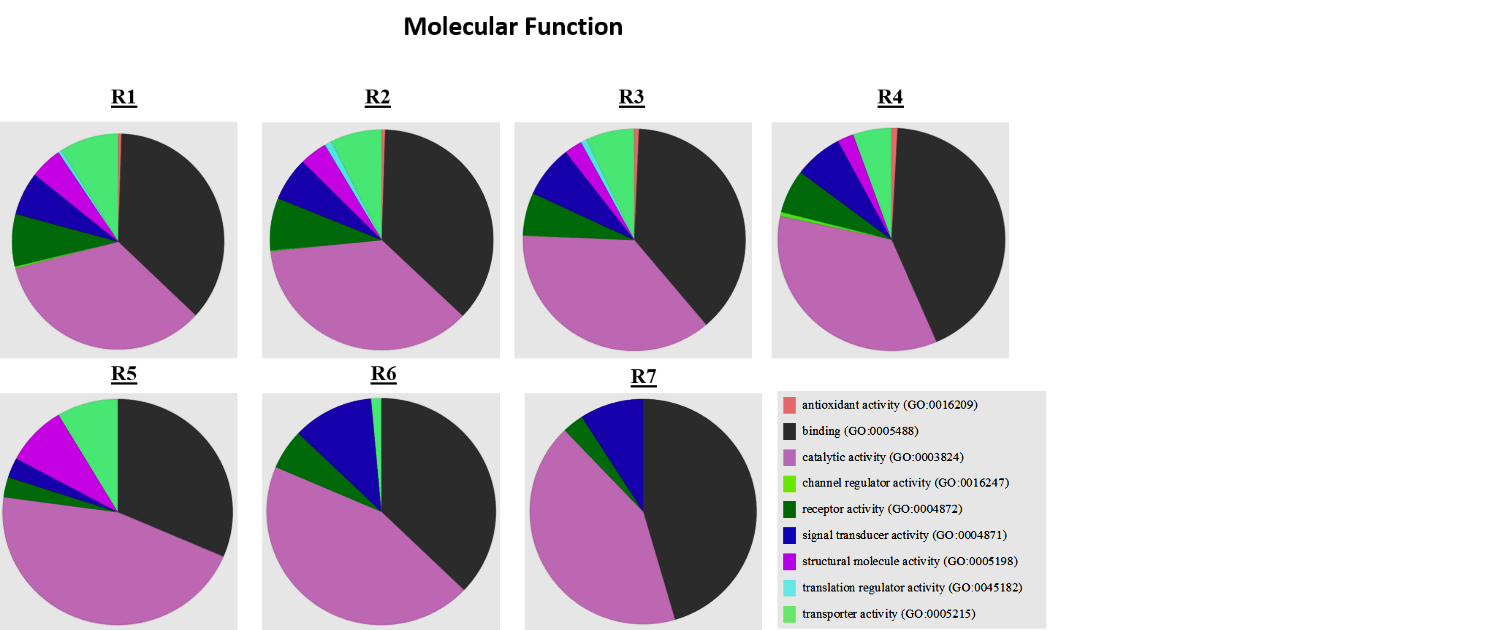

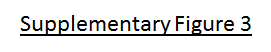


**Supplementary Figure 3. The molecular function (MF) distribution of R 1- 7 proteins.** The pie charts show the distribution of the molecular functions of the proteins belonging to R1 - R7 categories. Gene Ontology (GO) annotations have been used to define the involved pathways. Color legends aids in identification of the GO for the molecular functions.


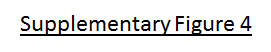


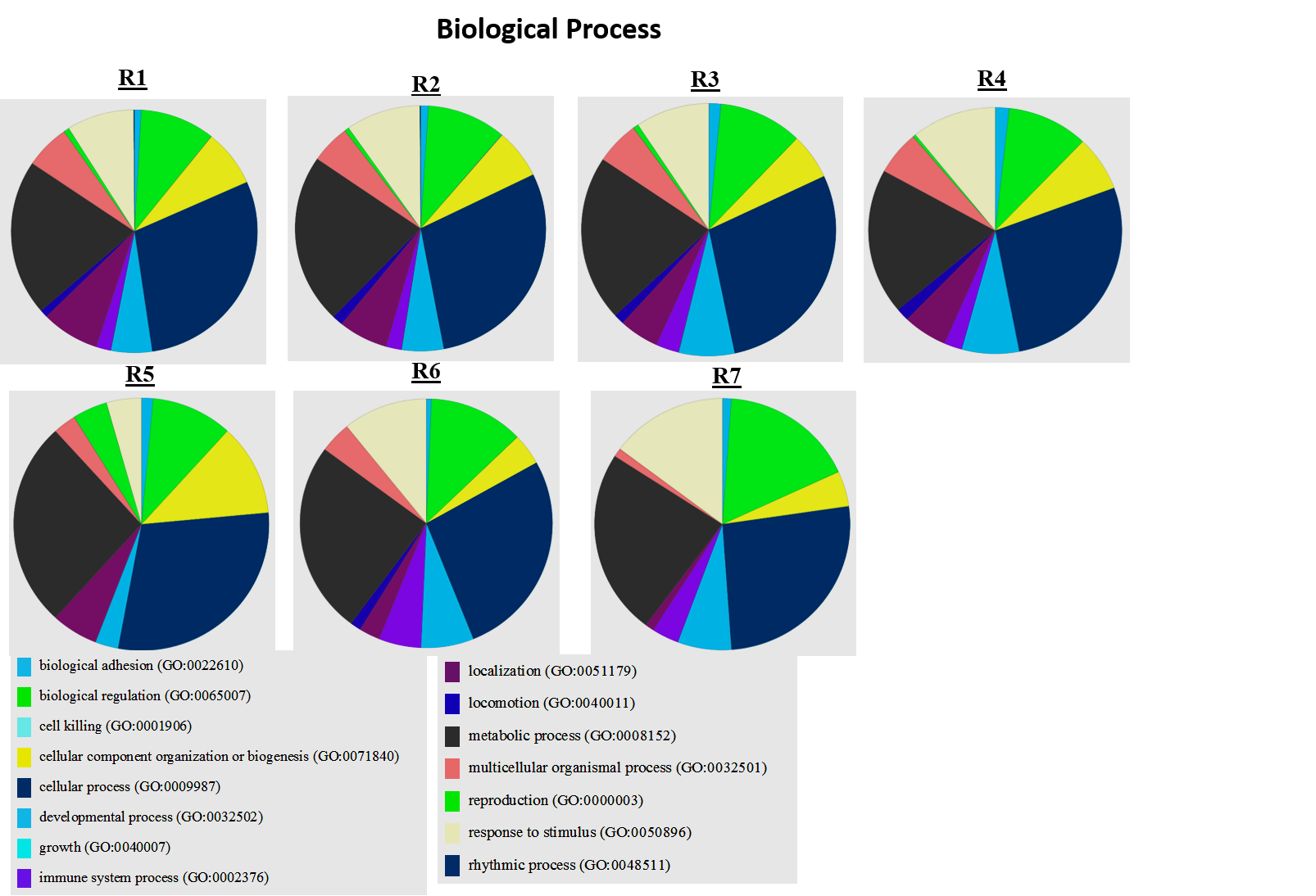


**Supplementary Figure 4. The biological process (BP) distribution of R 1- 7 proteins.** The pie charts show the distribution of the available biological processes of the proteins belonging to R1 - R7 categories. Gene Ontology (GO) annotations have been used to define the involved pathways. Color legends aids in identification of the GO for the biological processes.


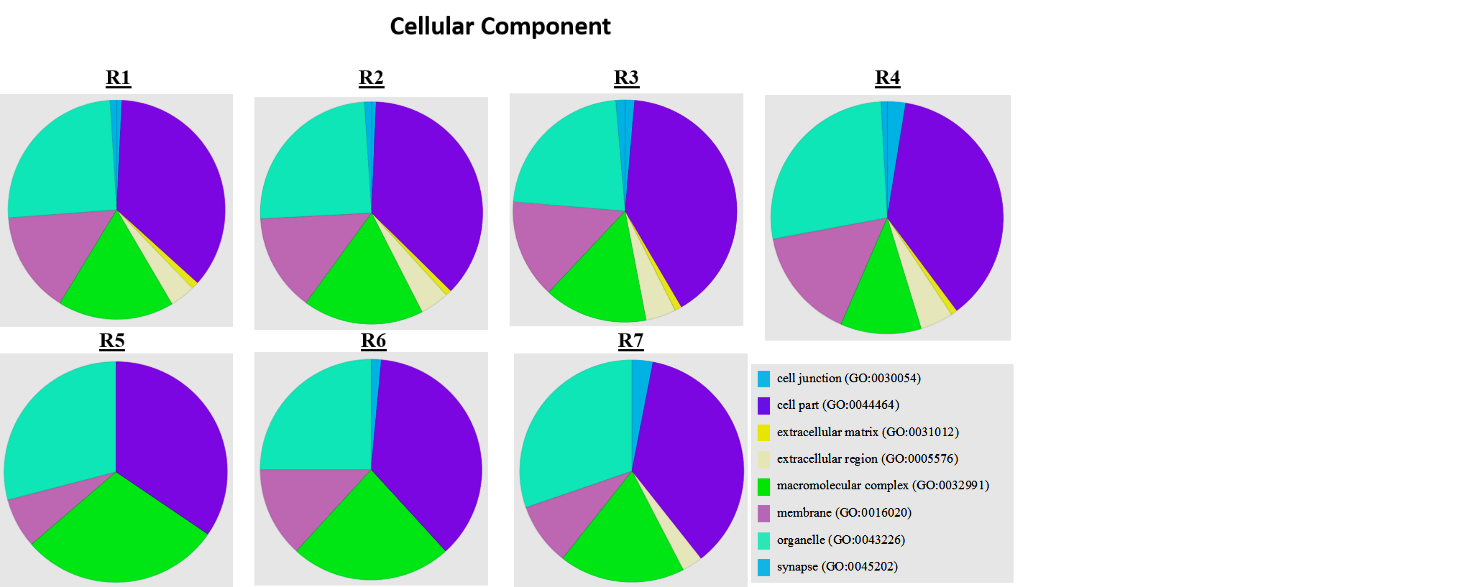

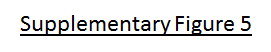


**Supplementary Figure 5. The cellular component (CC) distribution of R 1- 7 proteins.** The pie charts show the distribution of the cellular components of the proteins belonging to R1 - R7 categories. Gene Ontology (GO) annotations have been used to define the cellular components. Color legends aids in identification of the GO for the cellular components.


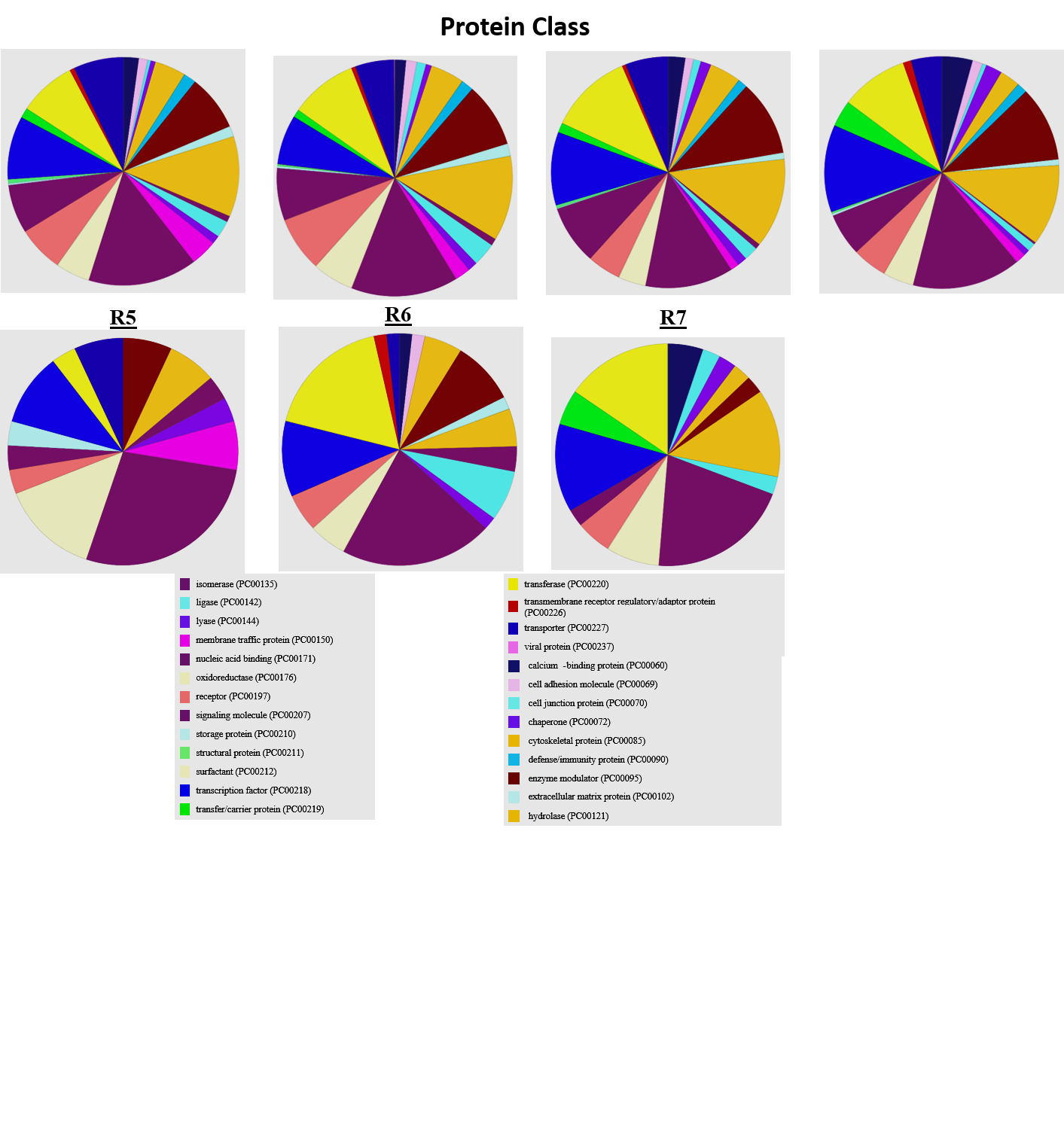

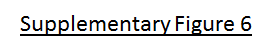


**Supplementary Figure 6. The protein class (PC) distribution of R 1- 7 proteins.** The pie charts show the distribution of the classes of the proteins belonging to R1 - R7 categories. Gene Ontology (GO) annotations have been used to define the protein classes. Color legends aids in identification of the GO for the classes of proteins.


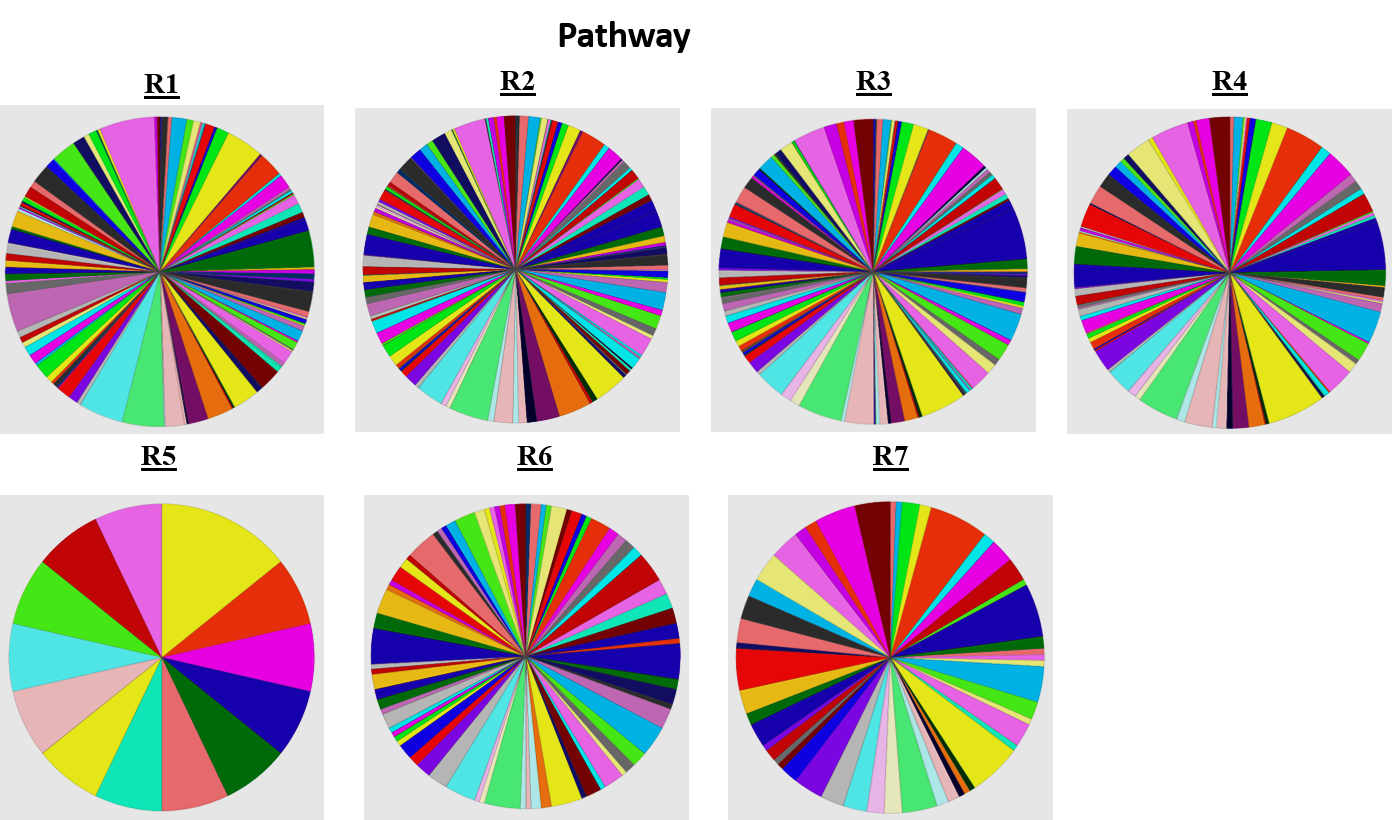

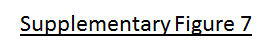


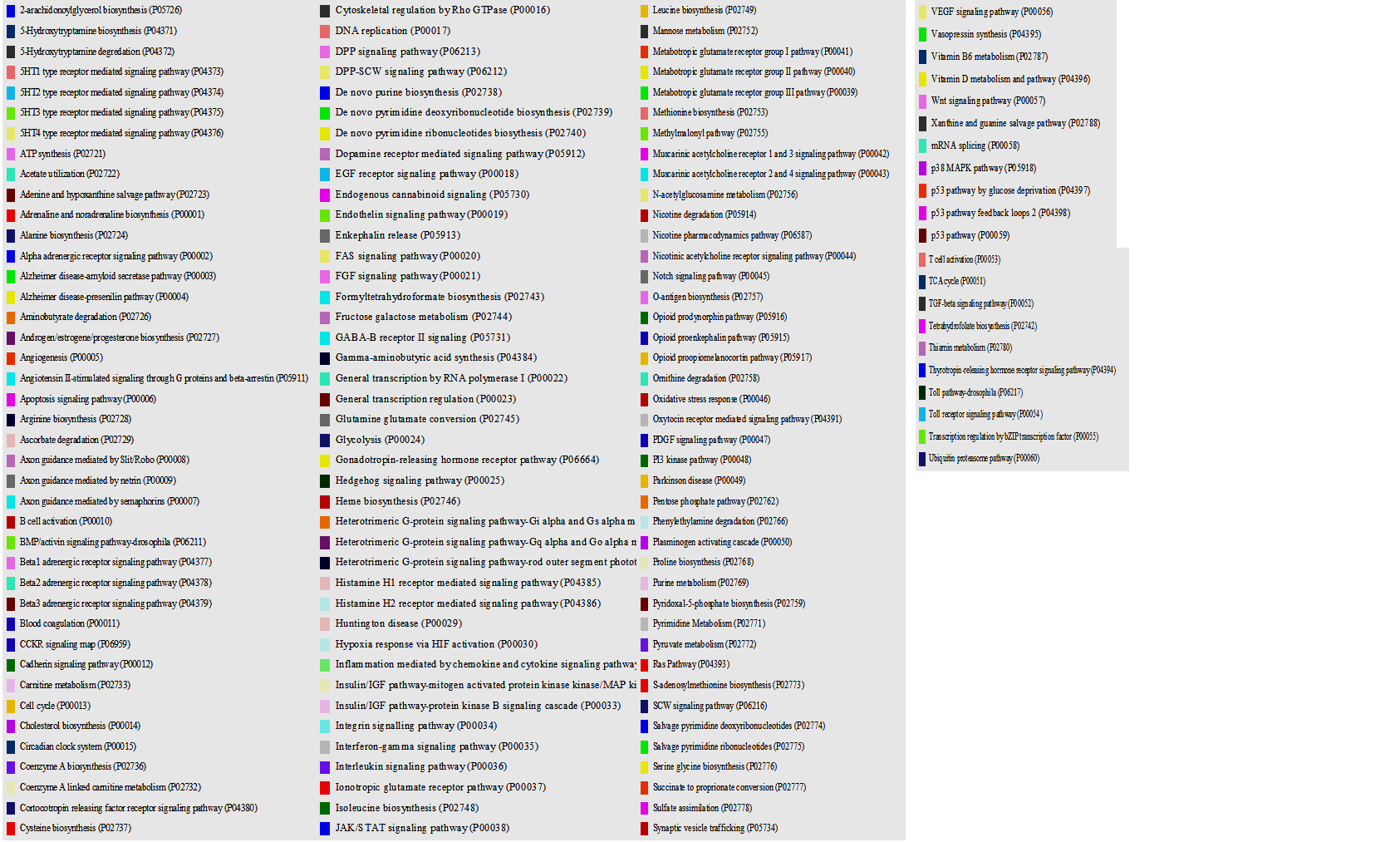


**Supplementary Figure 7. The pathway (PW) distribution of R 1- 7 proteins.** The pie charts show the distribution of the pathways involving the proteins belonging to R1 - R7 categories. Gene Ontology (GO) annotations have been used to define the involved pathways. Color legends aids in identification of the GO for the pathways.


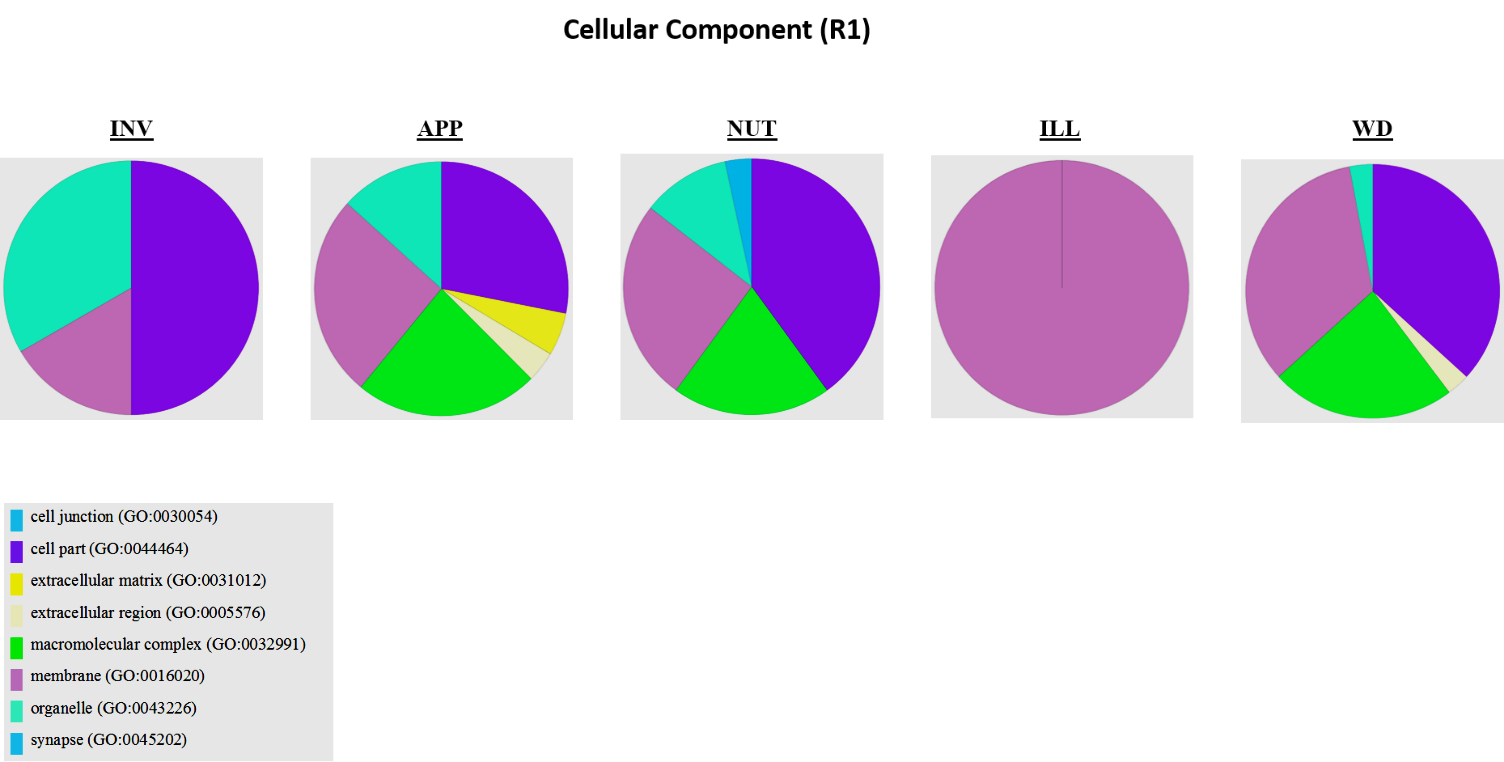

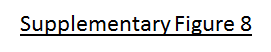


**Supplementary Figure 8. The cellular components distribution of R1 proteins for drug statuses.** The pie charts show the distribution of the cellular components of the proteins belonging to R1 for INV, APP, NUT, ILL and WD drug statuses. Gene Ontology (GO) annotations have been used to define the cellular components. Color legends aids in identification of the GO for the cellular components.


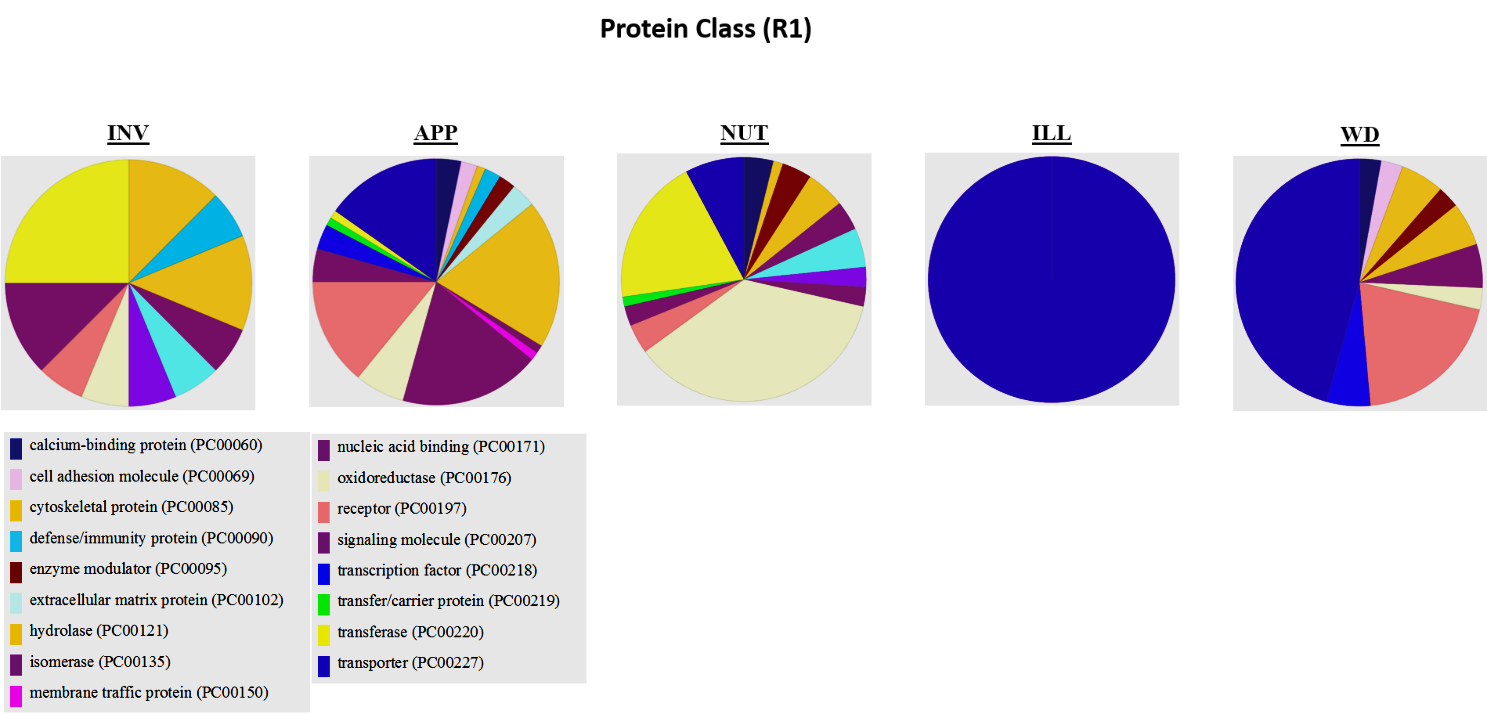

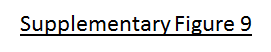


**Supplementary Figure 9. The protein class distribution of R1 proteins for drug statuses.** The pie charts show the distribution of the protein class of the proteins belonging to R1 for INV, APP, NUT, ILL and WD drug statuses. Gene Ontology (GO) annotations have been used to define the protein classes. Color legends aids in identification of the GO for the classes of proteins.


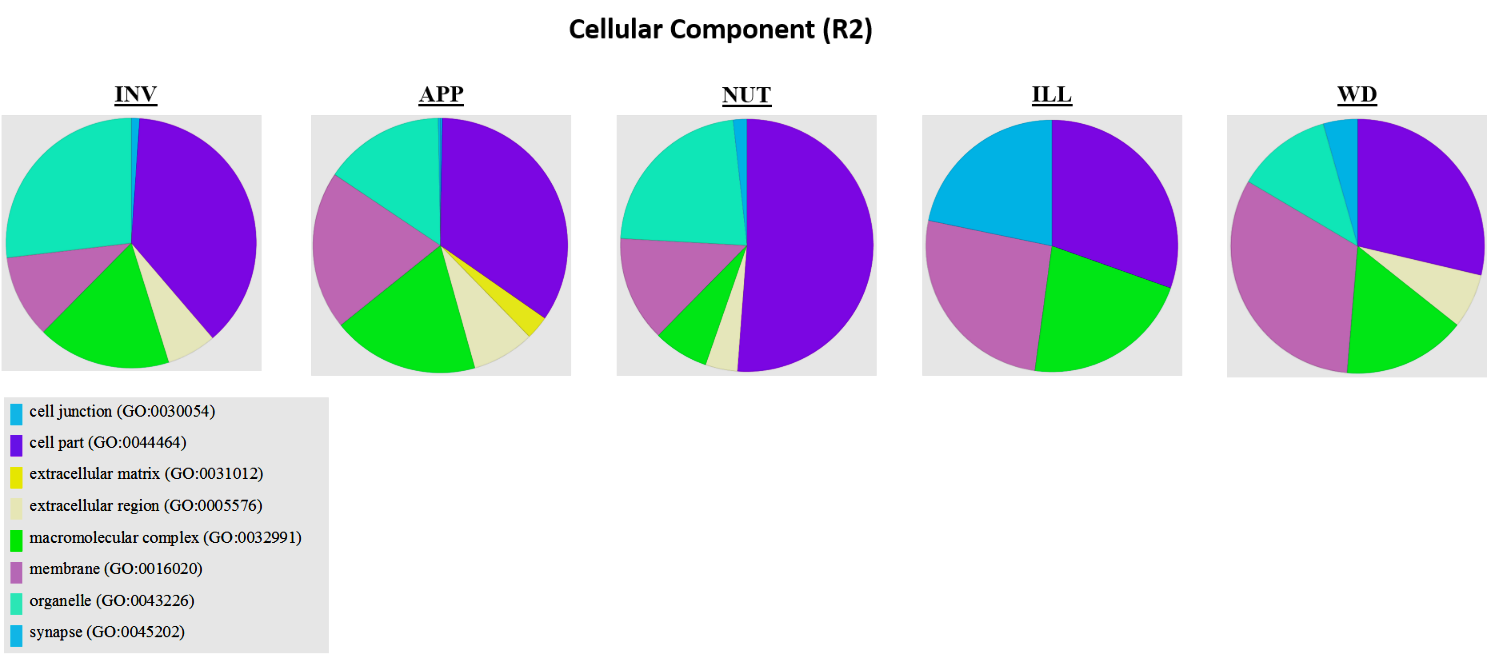

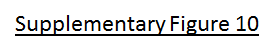


**Supplementary Figure 10. The cellular components distribution of R2 proteins for drug statuses.** The pie charts show the distribution of the cellular components of the proteins belonging to R2 for INV, APP, NUT, ILL and WD drug statuses. Gene Ontology (GO) annotations have been used to define the cellular components. Color legends aids in identification of the GO for the cellular components.


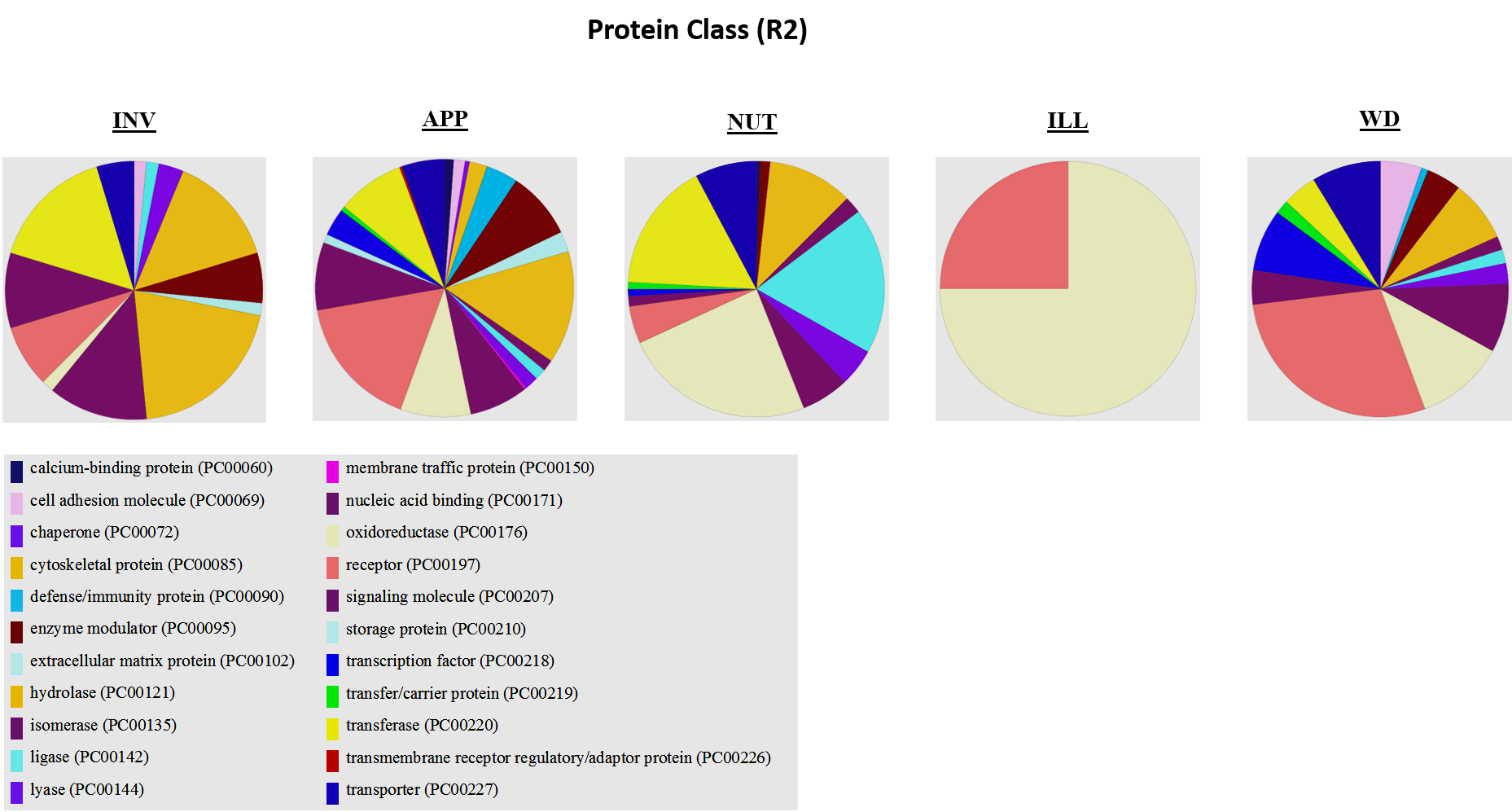

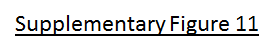


**Supplementary Figure 11. The protein class distribution of R2 proteins for drug statuses.** The pie charts show the distribution of the protein class of the proteins belonging to R2 for INV, APP, NUT, ILL and WD drug statuses. Gene Ontology (GO) annotations have been used to define the protein classes. Color legends aids in identification of the GO for the classes of proteins.


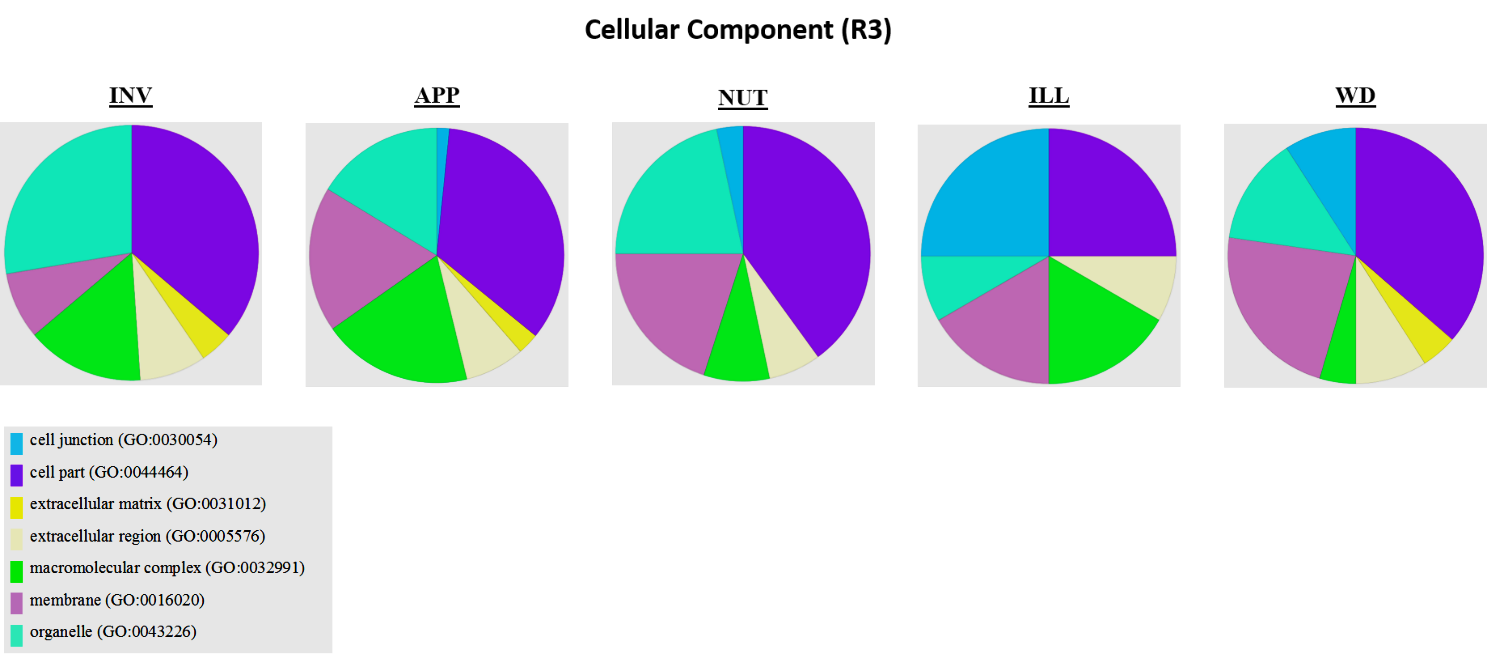

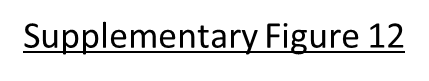


**Supplementary Figure 12. The cellular components distribution of R3 proteins for drug statuses.** The pie charts show the distribution of the cellular components of the proteins belonging to R3 for INV, APP, NUT, ILL and WD drug statuses. Gene Ontology (GO) annotations have been used to define the cellular components. Color legends aids in identification of the GO for the cellular components.


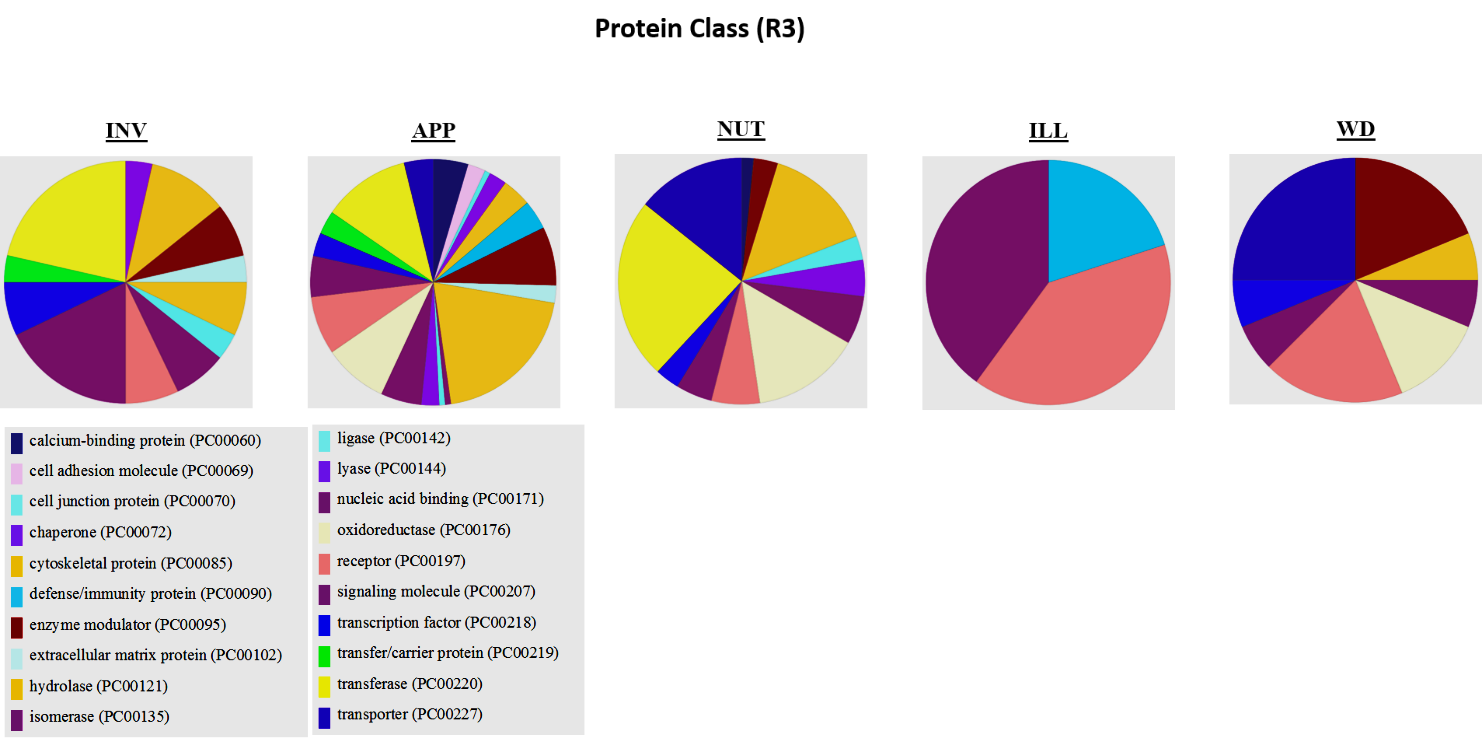

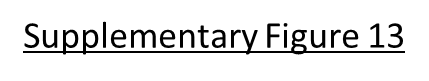


**Supplementary Figure 13. The protein class distribution of R3 proteins for drug statuses.** The pie charts show the distribution of the protein class of the proteins belonging to R3 for INV, APP, NUT, ILL and WD drug statuses. Gene Ontology (GO) annotations have been used to define the protein classes. Color legends aids in identification of the GO for the classes of proteins.


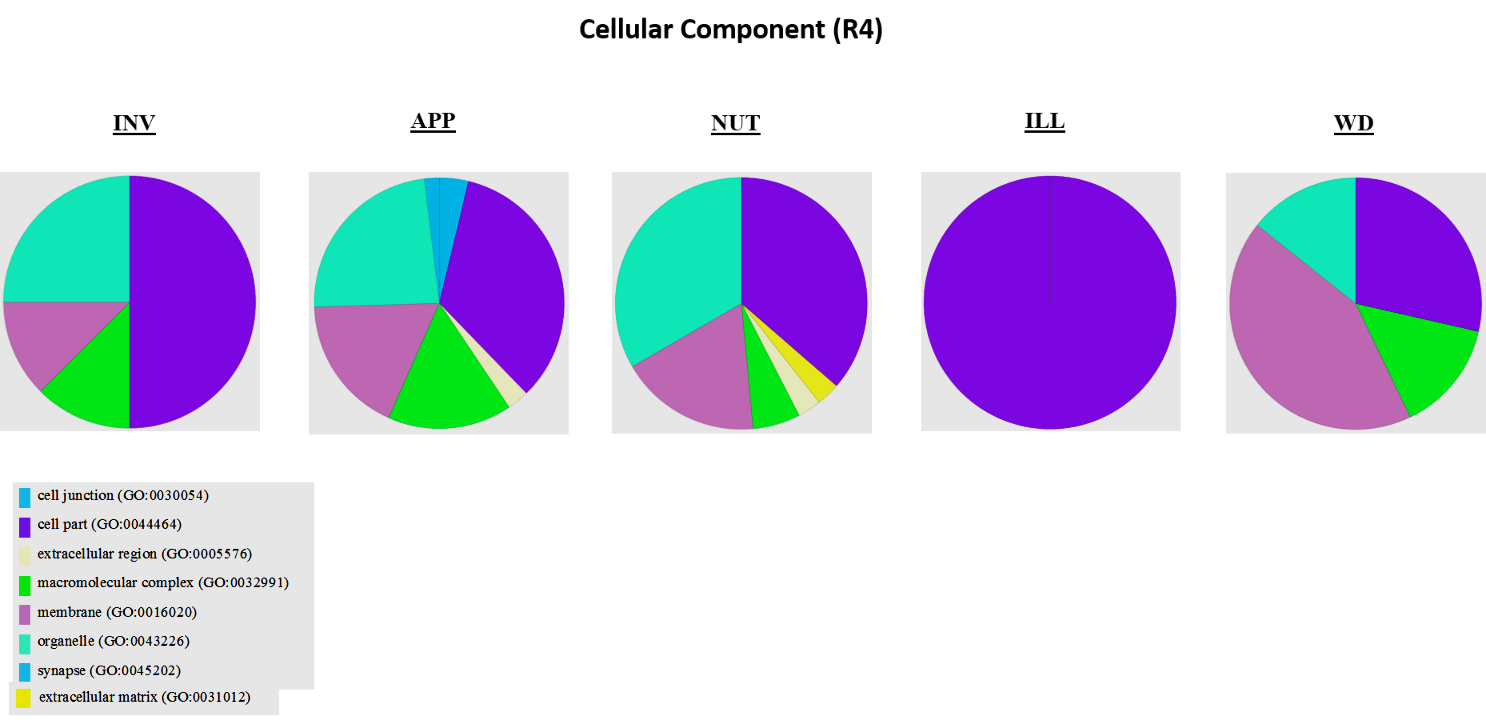

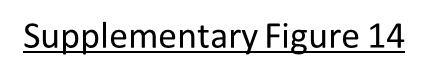


**Supplementary Figure 14. The cellular components distribution of R4 proteins for drug statuses.** The pie charts show the distribution of the cellular components of the proteins belonging to R4 for INV, APP, NUT, ILL and WD drug statuses. Gene Ontology (GO) annotations have been used to define the cellular components. Color legends aids in identification of the GO for the cellular components.


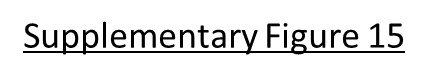


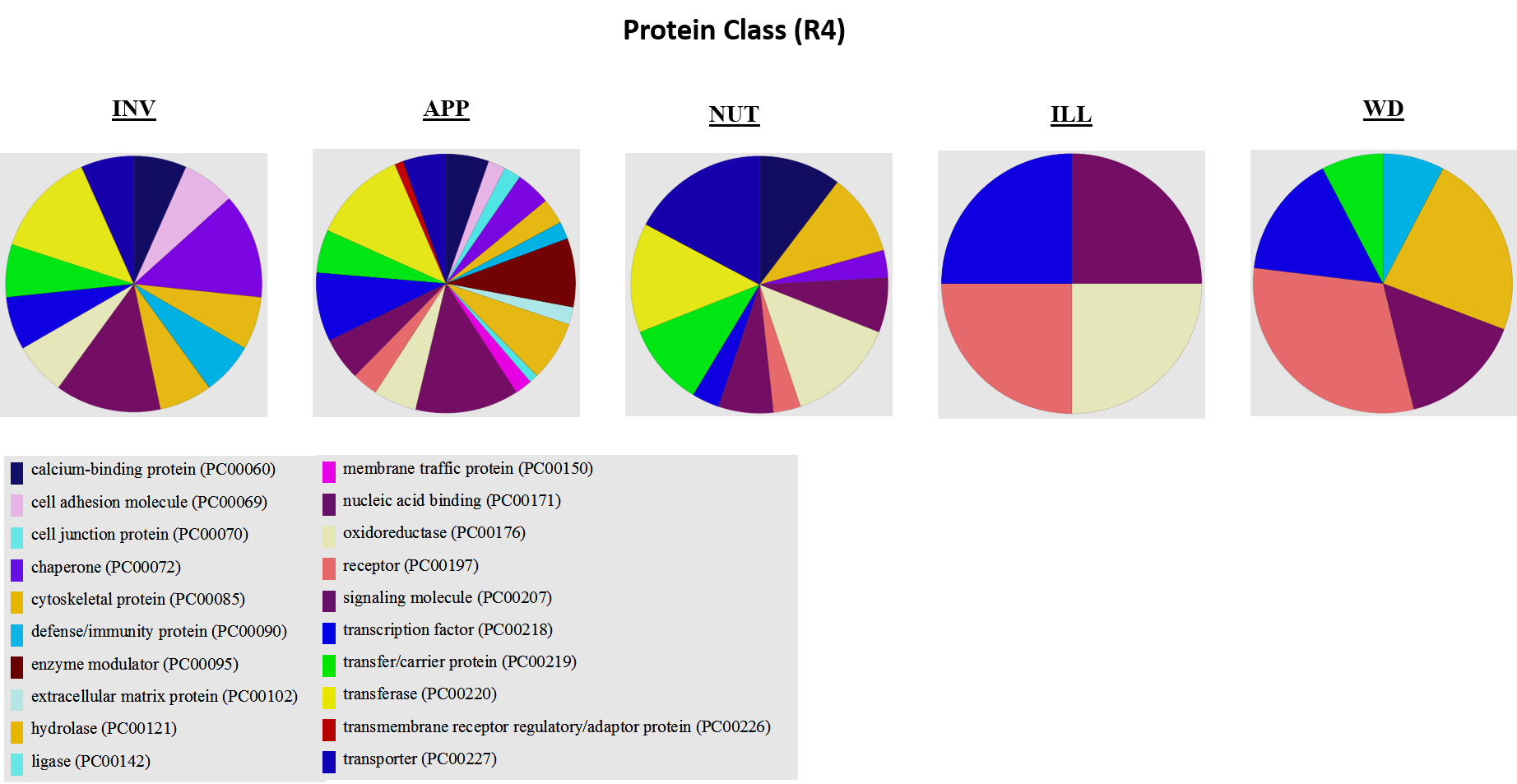


**Supplementary Figure 15. The protein class distribution of R4 proteins for drug statuses.** The pie charts show the distribution of the protein class of the proteins belonging to R4 for INV, APP, NUT, ILL and WD drug statuses. Gene Ontology (GO) annotations have been used to define the protein classes. Color legends aids in identification of the GO for the classes of proteins.


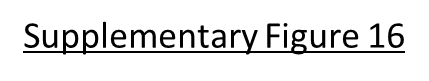


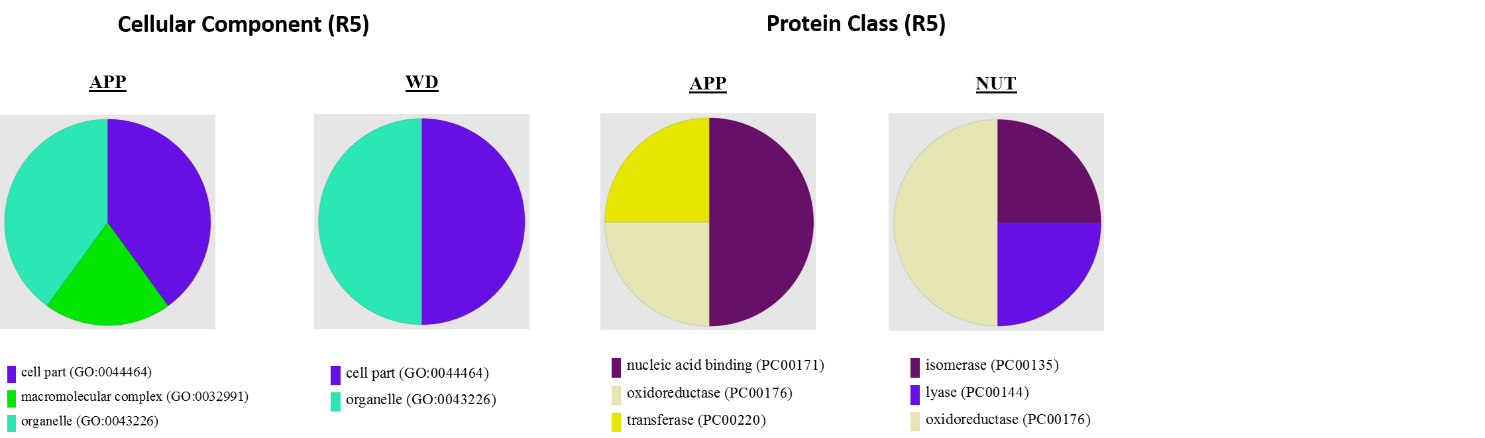


**Supplementary Figure 16. The cellular components and protein class distribution of R5 proteins for drug statuses.** The pie charts show the distribution of the cellular components and class of the proteins belonging to R5 for INV, APP, NUT and WD drug statuses. Gene Ontology (GO) annotations have been used to define the cellular components. Color legends aids in identification of the GO for the cellular components.


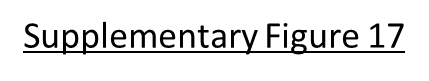


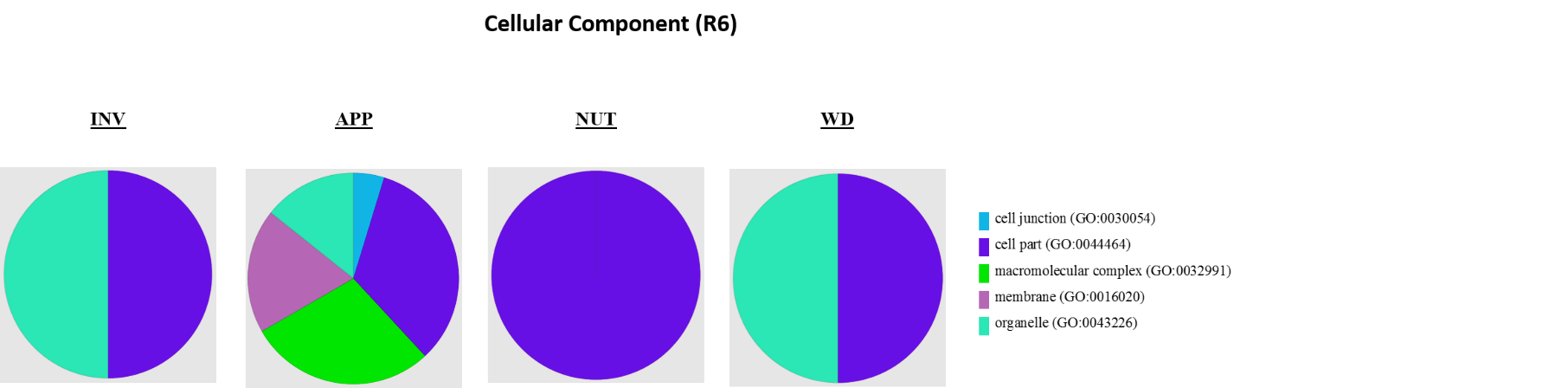


**Supplementary Figure 17. The cellular components distribution of R6 proteins for drug statuses.** The pie charts show the distribution of the cellular components of the proteins belonging to R6 for INV, APP, NUT and WD drug statuses. Gene Ontology (GO) annotations have been used to define the cellular components. Color legends aids in identification of the GO for the cellular components.


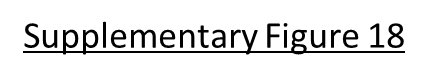


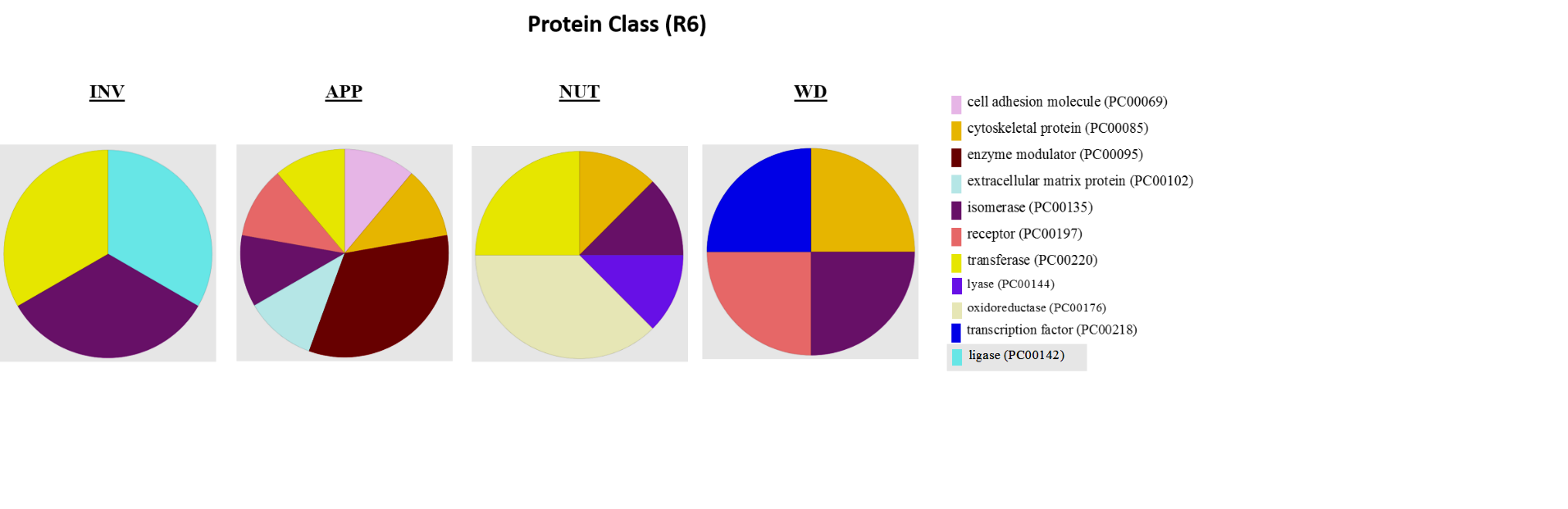


**Supplementary Figure 18. The protein class distribution of R6 proteins for drug statuses.** The pie charts show the distribution of the protein class of the proteins belonging to R6 for INV, APP, NUT and WD drug statuses. Gene Ontology (GO) annotations have been used to define the protein classes. Color legends aids in identification of the GO for the classes of proteins.


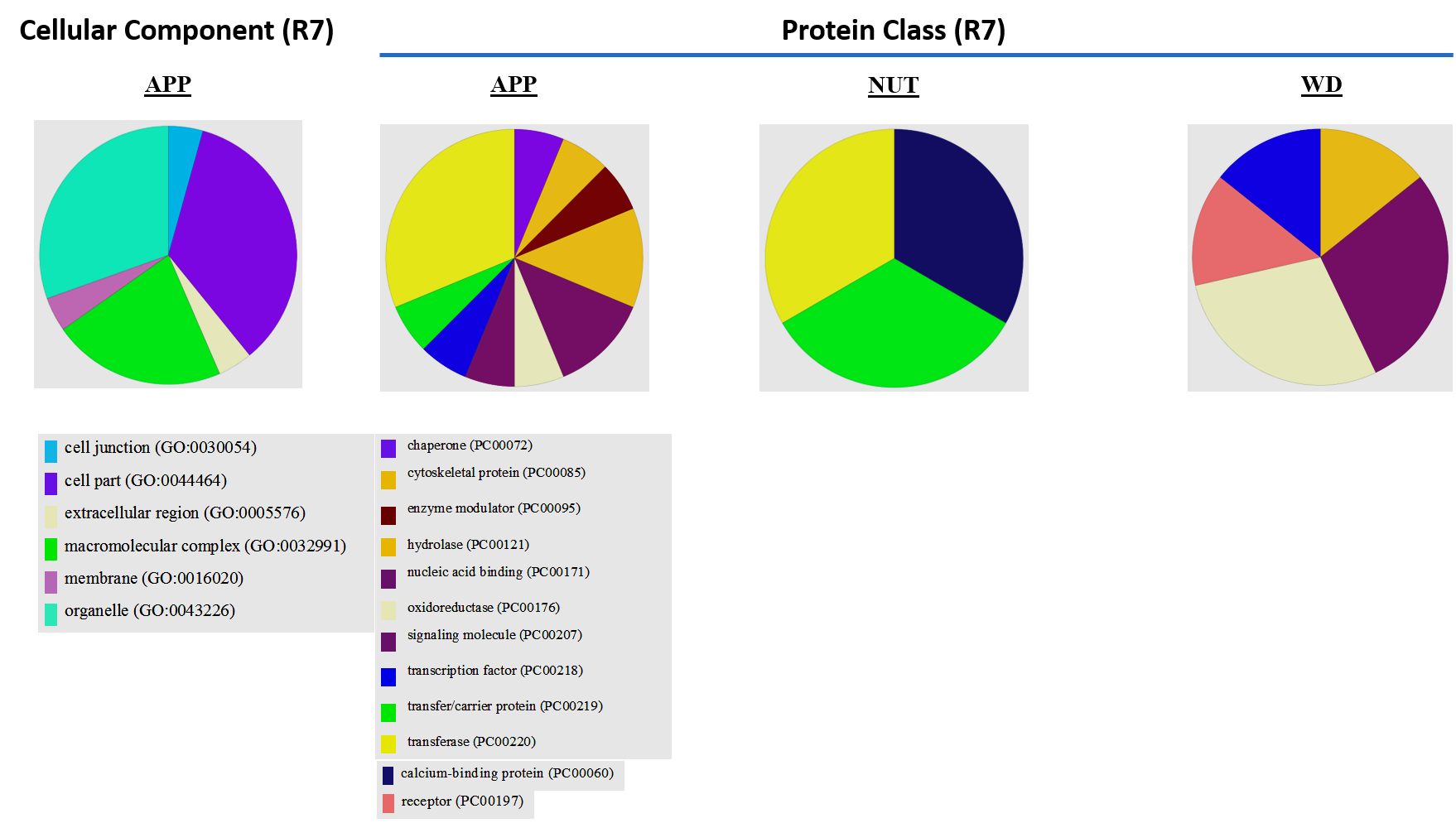

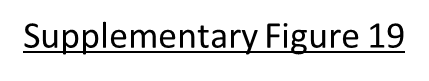


**Supplementary Figure 19. The cellular components and protein class distribution of R7 proteins for drug statuses.** The pie charts show the distribution of the cellular components and class of the proteins belonging to R7 for INV, APP, NUT and WD drug statuses. Gene Ontology (GO) annotations have been used to define the cellular components. Color legends aids in identification of the GO for the cellular components.
