# Supplementary material for: A side-effect free method for identifying cancer drug targets
Source: Sci Rep. 2018 Apr 27;8:6669. doi: 10.1038/s41598-018-25042-2 (PMC5923273; doi:10.1038/s41598-018-25042-2)
Supplement: Supplementary file 3 — Supplementary Data Statistics [file 41598_2018_25042_MOESM3_ESM.zip › 20180306 Centrality_Carto Output1.compressed.pdf]

```
*Nonparametric Tests: Independent Samples.
NPTESTS
  /INDEPENDENT TEST (BC_FR7n6 DC_FR7n6 EC_FR7n6) GROUP (FR7n6String)
  /MISSING SCOPE=ANALYSIS USERMISSING=EXCLUDE
  /CRITERIA ALPHA=0.05  CILEVEL=95.
```

Nonparametric Tests

```
[DataSet1] D:\20180306 Cancer Interactome Statistics\20180306 Centralit
rto.sav
```

Hypothesis Test Summary

	Null Hypothesis	Test	Sig.	Decision
1	The distribution of BC_FR7n6 is the same across categories of FR7n6String.	Independent-Samples Kruskal-Wallis Test	.000	Reject the null hypothesis.
2	The distribution of DC_FR7n6 is the same across categories of FR7n6String.	Independent-Samples Kruskal-Wallis Test	.000	Reject the null hypothesis.
3	The distribution of EC_FR7n6 is the same across categories of FR7n6String.	Independent-Samples Kruskal-Wallis Test	.000	Reject the null hypothesis.

Asymptotic significances are displayed. The significance level is .05.

```
*Nonparametric Tests: Independent Samples.
NPTESTS
  /INDEPENDENT TEST (BC_FR7n5 DC_FR7n5 EC_FR7n5) GROUP (FR7n5String)
  /MISSING SCOPE=ANALYSIS USERMISSING=EXCLUDE
  /CRITERIA ALPHA=0.05  CILEVEL=95.
```

Nonparametric Tests

Hypothesis Test Summary

	Null Hypothesis	Test	Sig.	Decision
1	The distribution of BC_FR7n5 is the same across categories of FR7n5String.	Independent-Samples Kruskal-Wallis Test	.000	Reject the null hypothesis.
2	The distribution of DC_FR7n5 is the same across categories of FR7n5String.	Independent-Samples Kruskal-Wallis Test	.000	Reject the null hypothesis.
3	The distribution of EC_FR7n5 is the same across categories of FR7n5String.	Independent-Samples Kruskal-Wallis Test	.000	Reject the null hypothesis.

Asymptotic significances are displayed. The significance level is .05.

```
*Nonparametric Tests: Independent Samples.
NPTESTS
  /INDEPENDENT TEST (BC_FR7n4 DC_FR7n4 EC_FR7n4) GROUP (FR7n4String)
  /MISSING SCOPE=ANALYSIS USERMISSING=EXCLUDE
  /CRITERIA ALPHA=0.05  CILEVEL=95.
```

Nonparametric Tests

Hypothesis Test Summary

	Null Hypothesis	Test	Sig.	Decision
1	The distribution of BC_FR7n4 is the same across categories of FR7n4String.	Independent-Samples Kruskal-Wallis Test	.000	Reject the null hypothesis.
2	The distribution of DC_FR7n4 is the same across categories of FR7n4String.	Independent-Samples Kruskal-Wallis Test	.000	Reject the null hypothesis.
3	The distribution of EC_FR7n4 is the same across categories of FR7n4String.	Independent-Samples Kruskal-Wallis Test	.000	Reject the null hypothesis.

Asymptotic significances are displayed. The significance level is .05.

```
*Nonparametric Tests: Independent Samples.
NPTESTS
  /INDEPENDENT TEST (BC_FR7n3 DC_FR7n3 EC_FR7n3) GROUP (FR7n3String)
  /MISSING SCOPE=ANALYSIS USERMISSING=EXCLUDE
  /CRITERIA ALPHA=0.05  CILEVEL=95.
```

Nonparametric Tests

Hypothesis Test Summary

	Null Hypothesis	Test	Sig.	Decision
1	The distribution of BC_FR7n3 is the same across categories of FR7n3String.	Independent-Samples Kruskal-Wallis Test	.000	Reject the null hypothesis.
2	The distribution of DC_FR7n3 is the same across categories of FR7n3String.	Independent-Samples Kruskal-Wallis Test	.000	Reject the null hypothesis.
3	The distribution of EC_FR7n3 is the same across categories of FR7n3String.	Independent-Samples Kruskal-Wallis Test	.000	Reject the null hypothesis.

Asymptotic significances are displayed. The significance level is .05.

```
*Nonparametric Tests: Independent Samples.
NPTESTS
  /INDEPENDENT TEST (BC_FR7n2 DC_FR7n2 EC_FR7n2) GROUP (FR7n2String)
  /MISSING SCOPE=ANALYSIS USERMISSING=EXCLUDE
  /CRITERIA ALPHA=0.05  CILEVEL=95.
```

Nonparametric Tests

Hypothesis Test Summary

	Null Hypothesis	Test	Sig.	Decision
1	The distribution of BC_FR7n2 is the same across categories of FR7n2String.	Independent-Samples Kruskal-Wallis Test	.000	Reject the null hypothesis.
2	The distribution of DC_FR7n2 is the same across categories of FR7n2String.	Independent-Samples Kruskal-Wallis Test	.000	Reject the null hypothesis.
3	The distribution of EC_FR7n2 is the same across categories of FR7n2String.	Independent-Samples Kruskal-Wallis Test	.000	Reject the null hypothesis.

Asymptotic significances are displayed. The significance level is .05.

```
*Nonparametric Tests: Independent Samples.
NPTESTS
  /INDEPENDENT TEST (BC_FR7n1 DC_FR7n1 EC_FR7n1) GROUP (FR7n1String)
  /MISSING SCOPE=ANALYSIS USERMISSING=EXCLUDE
  /CRITERIA ALPHA=0.05  CILEVEL=95.
```

Nonparametric Tests

Hypothesis Test Summary

	Null Hypothesis	Test	Sig.	Decision
1	The distribution of BC_FR7n1 is the same across categories of FR7n1String.	Independent-Samples Kruskal-Wallis Test	.000	Reject the null hypothesis.
2	The distribution of DC_FR7n1 is the same across categories of FR7n1String.	Independent-Samples Kruskal-Wallis Test	.000	Reject the null hypothesis.
3	The distribution of EC_FR7n1 is the same across categories of FR7n1String.	Independent-Samples Kruskal-Wallis Test	.000	Reject the null hypothesis.

Asymptotic significances are displayed. The significance level is .05.

```
*Nonparametric Tests: Independent Samples.
NPTESTS
  /INDEPENDENT TEST (BC_FR6n5 DC_FR6n5 EC_FR6n5) GROUP (FR6n5String)
  /MISSING SCOPE=ANALYSIS USERMISSING=EXCLUDE
  /CRITERIA ALPHA=0.05  CILEVEL=95.
```

Nonparametric Tests

Hypothesis Test Summary

	Null Hypothesis	Test	Sig.	Decision
1	The distribution of BC_FR6n5 is the same across categories of FR6n5String.	Independent-Samples Kruskal-Wallis Test	.006	Reject the null hypothesis.
2	The distribution of DC_FR6n5 is the same across categories of FR6n5String.	Independent-Samples Kruskal-Wallis Test	.184	Retain the null hypothesis.
3	The distribution of EC_FR6n5 is the same across categories of FR6n5String.	Independent-Samples Kruskal-Wallis Test	.001	Reject the null hypothesis.

Asymptotic significances are displayed. The significance level is .05.

```
*Nonparametric Tests: Independent Samples.
NPTESTS
  /INDEPENDENT TEST (BC_FR6n4 DC_FR6n4 EC_FR6n4) GROUP (FR6n4String)
  /MISSING SCOPE=ANALYSIS USERMISSING=EXCLUDE
  /CRITERIA ALPHA=0.05  CILEVEL=95.
```

Nonparametric Tests

Hypothesis Test Summary

	Null Hypothesis	Test	Sig.	Decision
1	The distribution of BC_FR6n4 is the same across categories of FR6n4String.	Independent-Samples Kruskal-Wallis Test	.000	Reject the null hypothesis.
2	The distribution of DC_FR6n4 is the same across categories of FR6n4String.	Independent-Samples Kruskal-Wallis Test	.000	Reject the null hypothesis.
3	The distribution of EC_FR6n4 is the same across categories of FR6n4String.	Independent-Samples Kruskal-Wallis Test	.049	Reject the null hypothesis.

Asymptotic significances are displayed. The significance level is .05.

```
*Nonparametric Tests: Independent Samples.
NPTESTS
  /INDEPENDENT TEST (BC_FR6n3 DC_FR6n3 EC_FR6n3) GROUP (FR6n3String)
  /MISSING SCOPE=ANALYSIS USERMISSING=EXCLUDE
  /CRITERIA ALPHA=0.05  CILEVEL=95.
```

Nonparametric Tests

Hypothesis Test Summary

	Null Hypothesis	Test	Sig.	Decision
1	The distribution of BC_FR6n3 is the same across categories of FR6n3String.	Independent-Samples Kruskal-Wallis Test	.000	Reject the null hypothesis.
2	The distribution of DC_FR6n3 is the same across categories of FR6n3String.	Independent-Samples Kruskal-Wallis Test	.000	Reject the null hypothesis.
3	The distribution of EC_FR6n3 is the same across categories of FR6n3String.	Independent-Samples Kruskal-Wallis Test	.000	Reject the null hypothesis.

Asymptotic significances are displayed. The significance level is .05.

```
*Nonparametric Tests: Independent Samples.
NPTESTS
  /INDEPENDENT TEST (BC_FR6n2 DC_FR6n2 EC_FR6n2) GROUP (FR6n2String)
  /MISSING SCOPE=ANALYSIS USERMISSING=EXCLUDE
  /CRITERIA ALPHA=0.05  CILEVEL=95.
```

Nonparametric Tests

Hypothesis Test Summary

	Null Hypothesis	Test	Sig.	Decision
1	The distribution of BC_FR6n2 is the same across categories of FR6n2String.	Independent-Samples Kruskal-Wallis Test	.000	Reject the null hypothesis.
2	The distribution of DC_FR6n2 is the same across categories of FR6n2String.	Independent-Samples Kruskal-Wallis Test	.000	Reject the null hypothesis.
3	The distribution of EC_FR6n2 is the same across categories of FR6n2String.	Independent-Samples Kruskal-Wallis Test	.000	Reject the null hypothesis.

Asymptotic significances are displayed. The significance level is .05.

```
*Nonparametric Tests: Independent Samples.
NPTESTS
  /INDEPENDENT TEST (BC_FR6n1 DC_FR6n1 EC_FR6n1) GROUP (FR6n1String)
  /MISSING SCOPE=ANALYSIS USERMISSING=EXCLUDE
  /CRITERIA ALPHA=0.05  CILEVEL=95.
```

Nonparametric Tests

Hypothesis Test Summary

	Null Hypothesis	Test	Sig.	Decision
1	The distribution of BC_FR6n1 is the same across categories of FR6n1String.	Independent-Samples Kruskal-Wallis Test	.000	Reject the null hypothesis.
2	The distribution of DC_FR6n1 is the same across categories of FR6n1String.	Independent-Samples Kruskal-Wallis Test	.000	Reject the null hypothesis.
3	The distribution of EC_FR6n1 is the same across categories of FR6n1String.	Independent-Samples Kruskal-Wallis Test	.000	Reject the null hypothesis.

Asymptotic significances are displayed. The significance level is .05.

```
*Nonparametric Tests: Independent Samples.
NPTESTS
  /INDEPENDENT TEST (BC_FR5n4 DC_FR5n4 EC_FR5n4) GROUP (FR5n4String)
  /MISSING SCOPE=ANALYSIS USERMISSING=EXCLUDE
  /CRITERIA ALPHA=0.05  CILEVEL=95.
```

Nonparametric Tests

Hypothesis Test Summary

	Null Hypothesis	Test	Sig.	Decision
1	The distribution of BC_FR5n4 is the same across categories of FR5n4String.	Independent-Samples Kruskal-Wallis Test	.559	Retain the null hypothesis.
2	The distribution of DC_FR5n4 is the same across categories of FR5n4String.	Independent-Samples Kruskal-Wallis Test	.000	Reject the null hypothesis.
3	The distribution of EC_FR5n4 is the same across categories of FR5n4String.	Independent-Samples Kruskal-Wallis Test	.005	Reject the null hypothesis.

Asymptotic significances are displayed. The significance level is .05.

```
*Nonparametric Tests: Independent Samples.
NPTESTS
  /INDEPENDENT TEST (BC_FR5n3 DC_FR5n3 EC_FR5n3) GROUP (FR5n3String)
  /MISSING SCOPE=ANALYSIS USERMISSING=EXCLUDE
  /CRITERIA ALPHA=0.05  CILEVEL=95.
```

Nonparametric Tests

Hypothesis Test Summary

	Null Hypothesis	Test	Sig.	Decision
1	The distribution of BC_FR5n3 is the same across categories of FR5n3String.	Independent-Samples Kruskal-Wallis Test	.000	Reject the null hypothesis.
2	The distribution of DC_FR5n3 is the same across categories of FR5n3String.	Independent-Samples Kruskal-Wallis Test	.000	Reject the null hypothesis.
3	The distribution of EC_FR5n3 is the same across categories of FR5n3String.	Independent-Samples Kruskal-Wallis Test	.712	Retain the null hypothesis.

Asymptotic significances are displayed. The significance level is .05.

```
*Nonparametric Tests: Independent Samples.
NPTESTS
  /INDEPENDENT TEST (BC_FR5n2 DC_FR5n2 EC_FR5n2) GROUP (FR5n2String)
  /MISSING SCOPE=ANALYSIS USERMISSING=EXCLUDE
  /CRITERIA ALPHA=0.05  CILEVEL=95.
```

Nonparametric Tests

Hypothesis Test Summary

	Null Hypothesis	Test	Sig.	Decision
1	The distribution of BC_FR5n2 is the same across categories of FR5n2String.	Independent-Samples Kruskal-Wallis Test	.000	Reject the null hypothesis.
2	The distribution of DC_FR5n2 is the same across categories of FR5n2String.	Independent-Samples Kruskal-Wallis Test	.000	Reject the null hypothesis.
3	The distribution of EC_FR5n2 is the same across categories of FR5n2String.	Independent-Samples Kruskal-Wallis Test	.010	Reject the null hypothesis.

Asymptotic significances are displayed. The significance level is .05.

```
*Nonparametric Tests: Independent Samples.
NPTESTS
  /INDEPENDENT TEST (BC_FR5n1 DC_FR5n1 EC_FR5n1) GROUP (FR5n1String)
  /MISSING SCOPE=ANALYSIS USERMISSING=EXCLUDE
  /CRITERIA ALPHA=0.05  CILEVEL=95.
```

Nonparametric Tests

Hypothesis Test Summary

	Null Hypothesis	Test	Sig.	Decision
1	The distribution of BC_FR5n1 is the same across categories of FR5n1String.	Independent-Samples Kruskal-Wallis Test	.000	Reject the null hypothesis.
2	The distribution of DC_FR5n1 is the same across categories of FR5n1String.	Independent-Samples Kruskal-Wallis Test	.000	Reject the null hypothesis.
3	The distribution of EC_FR5n1 is the same across categories of FR5n1String.	Independent-Samples Kruskal-Wallis Test	.000	Reject the null hypothesis.

Asymptotic significances are displayed. The significance level is .05.

```
*Nonparametric Tests: Independent Samples.
NPTESTS
  /INDEPENDENT TEST (BC_FR4n3 DC_FR4n3 EC_FR4n3) GROUP (FR4n3String)
  /MISSING SCOPE=ANALYSIS USERMISSING=EXCLUDE
  /CRITERIA ALPHA=0.05  CILEVEL=95.
```

Nonparametric Tests

Hypothesis Test Summary

	Null Hypothesis	Test	Sig.	Decision
1	The distribution of BC_FR4n3 is the same across categories of FR4n3String.	Independent-Samples Kruskal-Wallis Test	.000	Reject the null hypothesis.
2	The distribution of DC_FR4n3 is the same across categories of FR4n3String.	Independent-Samples Kruskal-Wallis Test	.000	Reject the null hypothesis.
3	The distribution of EC_FR4n3 is the same across categories of FR4n3String.	Independent-Samples Kruskal-Wallis Test	.000	Reject the null hypothesis.

Asymptotic significances are displayed. The significance level is .05.

```
*Nonparametric Tests: Independent Samples.
NPTESTS
  /INDEPENDENT TEST (BC_FR4n2 DC_FR4n2 EC_FR4n2) GROUP (FR4n2String)
  /MISSING SCOPE=ANALYSIS USERMISSING=EXCLUDE
  /CRITERIA ALPHA=0.05  CILEVEL=95.
```

Nonparametric Tests

Hypothesis Test Summary

	Null Hypothesis	Test	Sig.	Decision
1	The distribution of BC_FR4n2 is the same across categories of FR4n2String.	Independent-Samples Kruskal-Wallis Test	.000	Reject the null hypothesis.
2	The distribution of DC_FR4n2 is the same across categories of FR4n2String.	Independent-Samples Kruskal-Wallis Test	.000	Reject the null hypothesis.
3	The distribution of EC_FR4n2 is the same across categories of FR4n2String.	Independent-Samples Kruskal-Wallis Test	.000	Reject the null hypothesis.

Asymptotic significances are displayed. The significance level is .05.

```
*Nonparametric Tests: Independent Samples.
NPTESTS
  /INDEPENDENT TEST (BC_FR4n1 DC_FR4n1 EC_FR4n1) GROUP (FR4n1String)
  /MISSING SCOPE=ANALYSIS USERMISSING=EXCLUDE
  /CRITERIA ALPHA=0.05  CILEVEL=95.
```

Nonparametric Tests

Hypothesis Test Summary

	Null Hypothesis	Test	Sig.	Decision
1	The distribution of BC_FR4n1 is the same across categories of FR4n1String.	Independent-Samples Kruskal-Wallis Test	.000	Reject the null hypothesis.
2	The distribution of DC_FR4n1 is the same across categories of FR4n1String.	Independent-Samples Kruskal-Wallis Test	.000	Reject the null hypothesis.
3	The distribution of EC_FR4n1 is the same across categories of FR4n1String.	Independent-Samples Kruskal-Wallis Test	.000	Reject the null hypothesis.

Asymptotic significances are displayed. The significance level is .05.

```
*Nonparametric Tests: Independent Samples.
NPTESTS
  /INDEPENDENT TEST (BC_FR3n2 DC_FR3n2 EC_FR3n2) GROUP (FR3n2String)
  /MISSING SCOPE=ANALYSIS USERMISSING=EXCLUDE
  /CRITERIA ALPHA=0.05  CILEVEL=95.
```

Nonparametric Tests

Hypothesis Test Summary

	Null Hypothesis	Test	Sig.	Decision
1	The distribution of BC_FR3n2 is the same across categories of FR3n2String.	Independent-Samples Kruskal-Wallis Test	.000	Reject the null hypothesis.
2	The distribution of DC_FR3n2 is the same across categories of FR3n2String.	Independent-Samples Kruskal-Wallis Test	.000	Reject the null hypothesis.
3	The distribution of EC_FR3n2 is the same across categories of FR3n2String.	Independent-Samples Kruskal-Wallis Test	.000	Reject the null hypothesis.

Asymptotic significances are displayed. The significance level is .05.

```
*Nonparametric Tests: Independent Samples.
NPTESTS
  /INDEPENDENT TEST (BC_FR3n1 DC_FR3n1 EC_FR3n1) GROUP (FR3n1String)
  /MISSING SCOPE=ANALYSIS USERMISSING=EXCLUDE
  /CRITERIA ALPHA=0.05  CILEVEL=95.
```

Nonparametric Tests

Hypothesis Test Summary

	Null Hypothesis	Test	Sig.	Decision
1	The distribution of BC_FR3n1 is the same across categories of FR3n1String.	Independent-Samples Kruskal-Wallis Test	.000	Reject the null hypothesis.
2	The distribution of DC_FR3n1 is the same across categories of FR3n1String.	Independent-Samples Kruskal-Wallis Test	.000	Reject the null hypothesis.
3	The distribution of EC_FR3n1 is the same across categories of FR3n1String.	Independent-Samples Kruskal-Wallis Test	.000	Reject the null hypothesis.

Asymptotic significances are displayed. The significance level is .05.

```
*Nonparametric Tests: Independent Samples.
NPTESTS
  /INDEPENDENT TEST (BC_FR2n1 DC_FR2n1 EC_FR2n1) GROUP (FR2n1String)
  /MISSING SCOPE=ANALYSIS USERMISSING=EXCLUDE
  /CRITERIA ALPHA=0.05  CILEVEL=95.
```

Nonparametric Tests

Hypothesis Test Summary

	Null Hypothesis	Test	Sig.	Decision
1	The distribution of BC_FR2n1 is the same across categories of FR2n1String.	Independent-Samples Kruskal-Wallis Test	.000	Reject the null hypothesis.
2	The distribution of DC_FR2n1 is the same across categories of FR2n1String.	Independent-Samples Kruskal-Wallis Test	.000	Reject the null hypothesis.
3	The distribution of EC_FR2n1 is the same across categories of FR2n1String.	Independent-Samples Kruskal-Wallis Test	.000	Reject the null hypothesis.

Asymptotic significances are displayed. The significance level is .05.
