# Supplementary material for: A side-effect free method for identifying cancer drug targets
Source: Sci Rep. 2018 Apr 27;8:6669. doi: 10.1038/s41598-018-25042-2 (PMC5923273; doi:10.1038/s41598-018-25042-2)
Supplement: Supplementary file 3 — Supplementary Data Statistics [file 41598_2018_25042_MOESM3_ESM.zip › 20180306 Centrality_Drug Output1.compressed.pdf]

```
*Nonparametric Tests: Independent Samples.
NPTESTS
  /INDEPENDENT TEST (BC_WDnILL DC_WDnILL EC_WDnILL) GROUP (WDnILL_Strin
  /MISSING SCOPE=ANALYSIS USERMISSING=EXCLUDE
  /CRITERIA ALPHA=0.05  CILEVEL=95.
```

Nonparametric Tests

Hypothesis Test Summary

	Null Hypothesis	Test	Sig.	Decision
1	The distribution of BC_WDnILL is the same across categories of WDnILL_String.	Independent-Samples Kruskal-Wallis Test	.614	Retain the null hypothesis.
2	The distribution of DC_WDnILL is the same across categories of WDnILL_String.	Independent-Samples Kruskal-Wallis Test	.382	Retain the null hypothesis.
3	The distribution of EC_WDnILL is the same across categories of WDnILL_String.	Independent-Samples Kruskal-Wallis Test	.662	Retain the null hypothesis.

Asymptotic significances are displayed. The significance level is .05.

```
*Nonparametric Tests: Independent Samples.
NPTESTS
  /INDEPENDENT TEST (BC_WDnAPP DC_WDnAPP EC_WDnAPP) GROUP (WDnAPP_Strin
  /MISSING SCOPE=ANALYSIS USERMISSING=EXCLUDE
  /CRITERIA ALPHA=0.05  CILEVEL=95.
```

Nonparametric Tests

Hypothesis Test Summary

	Null Hypothesis	Test	Sig.	Decision
1	The distribution of BC_WDnAPP is the same across categories of WDnAPP_String.	Independent-Samples Kruskal-Wallis Test	.070	Retain the null hypothesis.
2	The distribution of DC_WDnAPP is the same across categories of WDnAPP_String.	Independent-Samples Kruskal-Wallis Test	.034	Reject the null hypothesis.
3	The distribution of EC_WDnAPP is the same across categories of WDnAPP_String.	Independent-Samples Kruskal-Wallis Test	.000	Reject the null hypothesis.

Asymptotic significances are displayed. The significance level is .05.

```
*Nonparametric Tests: Independent Samples.
NPTESTS
  /INDEPENDENT TEST (BC_WDnINV DC_WDnINV EC_WDnINV) GROUP (WDnINV_Strin
  /MISSING SCOPE=ANALYSIS USERMISSING=EXCLUDE
  /CRITERIA ALPHA=0.05  CILEVEL=95.
```

Nonparametric Tests

Hypothesis Test Summary

	Null Hypothesis	Test	Sig.	Decision
1	The distribution of BC_WDnINV is the same across categories of WDnINV_String.	Independent-Samples Kruskal-Wallis Test	.173	Retain the null hypothesis.
2	The distribution of DC_WDnINV is the same across categories of WDnINV_String.	Independent-Samples Kruskal-Wallis Test	.011	Reject the null hypothesis.
3	The distribution of EC_WDnINV is the same across categories of WDnINV_String.	Independent-Samples Kruskal-Wallis Test	.000	Reject the null hypothesis.

Asymptotic significances are displayed. The significance level is .05.

```
*Nonparametric Tests: Independent Samples.
NPTESTS
  /INDEPENDENT TEST (BC_WDnNUT DC_WDnNUT EC_WDnNUT) GROUP (WDnNUT_Strin
  /MISSING SCOPE=ANALYSIS USERMISSING=EXCLUDE
  /CRITERIA ALPHA=0.05  CILEVEL=95.
```

Nonparametric Tests

Hypothesis Test Summary

	Null Hypothesis	Test	Sig.	Decision
1	The distribution of BC_WDnNUT is the same across categories of WDnNUT_String.	Independent-Samples Kruskal-Wallis Test	.473	Retain the null hypothesis.
2	The distribution of DC_WDnNUT is the same across categories of WDnNUT_String.	Independent-Samples Kruskal-Wallis Test	.837	Retain the null hypothesis.
3	The distribution of EC_WDnNUT is the same across categories of WDnNUT_String.	Independent-Samples Kruskal-Wallis Test	.000	Reject the null hypothesis.

Asymptotic significances are displayed. The significance level is .05.

```
*Nonparametric Tests: Independent Samples.
NPTESTS
  /INDEPENDENT TEST (BC_WDnEXP DC_WDnEXP EC_WDnEXP) GROUP (WDnEXP_Strin
  /MISSING SCOPE=ANALYSIS USERMISSING=EXCLUDE
  /CRITERIA ALPHA=0.05  CILEVEL=95.
```

Nonparametric Tests

Hypothesis Test Summary

	Null Hypothesis	Test	Sig.	Decision
1	The distribution of BC_WDnEXP is the same across categories of WDnEXP_String.	Independent-Samples Kruskal-Wallis Test	.442	Retain the null hypothesis.
2	The distribution of DC_WDnEXP is the same across categories of WDnEXP_String.	Independent-Samples Kruskal-Wallis Test	.550	Retain the null hypothesis.
3	The distribution of EC_WDnEXP is the same across categories of WDnEXP_String.	Independent-Samples Kruskal-Wallis Test	.106	Retain the null hypothesis.

Asymptotic significances are displayed. The significance level is .05.

```
*Nonparametric Tests: Independent Samples.
NPTESTS
  /INDEPENDENT TEST (BC_WDnNA DC_WDnNA EC_WDnNA) GROUP (WDnNA_String)
  /MISSING SCOPE=ANALYSIS USERMISSING=EXCLUDE
  /CRITERIA ALPHA=0.05  CILEVEL=95.
```

Nonparametric Tests

Hypothesis Test Summary

	Null Hypothesis	Test	Sig.	Decision
1	The distribution of BC_WDnNA is the same across categories of WDnNA_String.	Independent-Samples Kruskal-Wallis Test	.000	Reject the null hypothesis.
2	The distribution of DC_WDnNA is the same across categories of WDnNA_String.	Independent-Samples Kruskal-Wallis Test	.000	Reject the null hypothesis.
3	The distribution of EC_WDnNA is the same across categories of WDnNA_String.	Independent-Samples Kruskal-Wallis Test	.000	Reject the null hypothesis.

Asymptotic significances are displayed. The significance level is .05.

```
*Nonparametric Tests: Independent Samples.
NPTESTS
  /INDEPENDENT TEST (BC_ILLnAPP DC_ILLnAPP EC_ILLnAPP) GROUP (ILLnAPP_St
g)
  /MISSING SCOPE=ANALYSIS USERMISSING=EXCLUDE
  /CRITERIA ALPHA=0.05  CILEVEL=95.
```

Nonparametric Tests

Hypothesis Test Summary

	Null Hypothesis	Test	Sig.	Decision
1	The distribution of BC_ILLnAPP is the same across categories of ILLnAPP_String.	Independent-Samples Kruskal-Wallis Test	.872	Retain the null hypothesis.
2	The distribution of DC_ILLnAPP is the same across categories of ILLnAPP_String.	Independent-Samples Kruskal-Wallis Test	.117	Retain the null hypothesis.
3	The distribution of EC_ILLnAPP is the same across categories of ILLnAPP_String.	Independent-Samples Kruskal-Wallis Test	.480	Retain the null hypothesis.

Asymptotic significances are displayed. The significance level is .05.

```
*Nonparametric Tests: Independent Samples.
NPTESTS
  /INDEPENDENT TEST (BC_ILLnINV DC_ILLnINV EC_ILLnINV) GROUP (ILLnINV_St
g)
  /MISSING SCOPE=ANALYSIS USERMISSING=EXCLUDE
  /CRITERIA ALPHA=0.05  CILEVEL=95.
```

Nonparametric Tests

Hypothesis Test Summary

	Null Hypothesis	Test	Sig.	Decision
1	The distribution of BC_ILLnINV is the same across categories of ILLnINV_String.	Independent-Samples Kruskal-Wallis Test	.817	Retain the null hypothesis.
2	The distribution of DC_ILLnINV is the same across categories of ILLnINV_String.	Independent-Samples Kruskal-Wallis Test	.071	Retain the null hypothesis.
3	The distribution of EC_ILLnINV is the same across categories of ILLnINV_String.	Independent-Samples Kruskal-Wallis Test	.111	Retain the null hypothesis.

Asymptotic significances are displayed. The significance level is .05.

```
*Nonparametric Tests: Independent Samples.
NPTESTS
  /INDEPENDENT TEST (BC_ILLnNUT DC_ILLnNUT EC_ILLnNUT) GROUP (ILLnNUT_St
g)
  /MISSING SCOPE=ANALYSIS USERMISSING=EXCLUDE
  /CRITERIA ALPHA=0.05  CILEVEL=95.
```

Nonparametric Tests

Hypothesis Test Summary

	Null Hypothesis	Test	Sig.	Decision
1	The distribution of BC_ILLnNUT is the same across categories of ILLnNUT_String.	Independent-Samples Kruskal-Wallis Test	.802	Retain the null hypothesis.
2	The distribution of DC_ILLnNUT is the same across categories of ILLnNUT_String.	Independent-Samples Kruskal-Wallis Test	.448	Retain the null hypothesis.
3	The distribution of EC_ILLnNUT is the same across categories of ILLnNUT_String.	Independent-Samples Kruskal-Wallis Test	.003	Reject the null hypothesis.

Asymptotic significances are displayed. The significance level is .05.

```
*Nonparametric Tests: Independent Samples.
NPTESTS
  /INDEPENDENT TEST (BC_ILLnEXP DC_ILLnEXP EC_ILLnEXP) GROUP (ILLnEXP_St
g)
  /MISSING SCOPE=ANALYSIS USERMISSING=EXCLUDE
  /CRITERIA ALPHA=0.05  CILEVEL=95.
```

Nonparametric Tests

Hypothesis Test Summary

	Null Hypothesis	Test	Sig.	Decision
1	The distribution of BC_ILLnEXP is the same across categories of ILLnEXP_String.	Independent-Samples Kruskal-Wallis Test	.872	Retain the null hypothesis.
2	The distribution of DC_ILLnEXP is the same across categories of ILLnEXP_String.	Independent-Samples Kruskal-Wallis Test	.336	Retain the null hypothesis.
3	The distribution of EC_ILLnEXP is the same across categories of ILLnEXP_String.	Independent-Samples Kruskal-Wallis Test	.969	Retain the null hypothesis.

Asymptotic significances are displayed. The significance level is .05.

```
*Nonparametric Tests: Independent Samples.
NPTESTS
  /INDEPENDENT TEST (BC_ILLnNA DC_ILLnNA EC_ILLnNA) GROUP (ILLnNA_Strin
  /MISSING SCOPE=ANALYSIS USERMISSING=EXCLUDE
  /CRITERIA ALPHA=0.05  CILEVEL=95.
```

Nonparametric Tests

Hypothesis Test Summary

	Null Hypothesis	Test	Sig.	Decision
1	The distribution of BC_ILLnNA is the same across categories of ILLnNA_String.	Independent-Samples Kruskal-Wallis Test	.014	Reject the null hypothesis.
2	The distribution of DC_ILLnNA is the same across categories of ILLnNA_String.	Independent-Samples Kruskal-Wallis Test	.037	Reject the null hypothesis.
3	The distribution of EC_ILLnNA is the same across categories of ILLnNA_String.	Independent-Samples Kruskal-Wallis Test	.026	Reject the null hypothesis.

Asymptotic significances are displayed. The significance level is .05.

```
*Nonparametric Tests: Independent Samples.
NPTESTS
  /INDEPENDENT TEST (BC_APPnINV DC_APPnINV EC_APPnINV) GROUP (APPnINV_St
g)
  /MISSING SCOPE=ANALYSIS USERMISSING=EXCLUDE
  /CRITERIA ALPHA=0.05  CILEVEL=95.
```

Nonparametric Tests

Hypothesis Test Summary

	Null Hypothesis	Test	Sig.	Decision
1	The distribution of BC_APPnINV is the same across categories of APPnINV_String.	Independent-Samples Kruskal-Wallis Test	.949	Retain the null hypothesis.
2	The distribution of DC_APPnINV is the same across categories of APPnINV_String.	Independent-Samples Kruskal-Wallis Test	.193	Retain the null hypothesis.
3	The distribution of EC_APPnINV is the same across categories of APPnINV_String.	Independent-Samples Kruskal-Wallis Test	.042	Reject the null hypothesis.

Asymptotic significances are displayed. The significance level is .05.

```
*Nonparametric Tests: Independent Samples.
NPTESTS
  /INDEPENDENT TEST (BC_APPnNUT DC_APPnNUT EC_APPnNUT) GROUP (APPnNUT_St
g)
  /MISSING SCOPE=ANALYSIS USERMISSING=EXCLUDE
  /CRITERIA ALPHA=0.05  CILEVEL=95.
```

Nonparametric Tests

Hypothesis Test Summary

	Null Hypothesis	Test	Sig.	Decision
1	The distribution of BC_APPnNUT is the same across categories of APPnNUT_String.	Independent-Samples Kruskal-Wallis Test	.097	Retain the null hypothesis.
2	The distribution of DC_APPnNUT is the same across categories of APPnNUT_String.	Independent-Samples Kruskal-Wallis Test	.001	Reject the null hypothesis.
3	The distribution of EC_APPnNUT is the same across categories of APPnNUT_String.	Independent-Samples Kruskal-Wallis Test	.000	Reject the null hypothesis.

Asymptotic significances are displayed. The significance level is .05.

```
*Nonparametric Tests: Independent Samples.
NPTESTS
  /INDEPENDENT TEST (BC_APPnEXP DC_APPnEXP EC_APPnEXP) GROUP (APPnEXP_St
g)
  /MISSING SCOPE=ANALYSIS USERMISSING=EXCLUDE
  /CRITERIA ALPHA=0.05  CILEVEL=95.
```

Nonparametric Tests

Hypothesis Test Summary

	Null Hypothesis	Test	Sig.	Decision
1	The distribution of BC_APPnEXP is the same across categories of APPnEXP_String.	Independent-Samples Kruskal-Wallis Test	.268	Retain the null hypothesis.
2	The distribution of DC_APPnEXP is the same across categories of APPnEXP_String.	Independent-Samples Kruskal-Wallis Test	.107	Retain the null hypothesis.
3	The distribution of EC_APPnEXP is the same across categories of APPnEXP_String.	Independent-Samples Kruskal-Wallis Test	.017	Reject the null hypothesis.

Asymptotic significances are displayed. The significance level is .05.

```
*Nonparametric Tests: Independent Samples.
NPTESTS
  /INDEPENDENT TEST (BC_APPnNA DC_APPnNA EC_APPnNA) GROUP (APPnNA_Strin
  /MISSING SCOPE=ANALYSIS USERMISSING=EXCLUDE
  /CRITERIA ALPHA=0.05  CILEVEL=95.
```

Nonparametric Tests

Hypothesis Test Summary

	Null Hypothesis	Test	Sig.	Decision
1	The distribution of BC_APPnNA is the same across categories of APPnNA_String.	Independent-Samples Kruskal-Wallis Test	.000	Reject the null hypothesis.
2	The distribution of DC_APPnNA is the same across categories of APPnNA_String.	Independent-Samples Kruskal-Wallis Test	.000	Reject the null hypothesis.
3	The distribution of EC_APPnNA is the same across categories of APPnNA_String.	Independent-Samples Kruskal-Wallis Test	.000	Reject the null hypothesis.

Asymptotic significances are displayed. The significance level is .05.

```
*Nonparametric Tests: Independent Samples.
NPTESTS
  /INDEPENDENT TEST (BC_INVnNUT DC_INVnNUT EC_INVnNUT) GROUP (INVnNUT_St
g)
  /MISSING SCOPE=ANALYSIS USERMISSING=EXCLUDE
  /CRITERIA ALPHA=0.05  CILEVEL=95.
```

Nonparametric Tests

Hypothesis Test Summary

	Null Hypothesis	Test	Sig.	Decision
1	The distribution of BC_INVnNUT is the same across categories of INVnNUT_String.	Independent-Samples Kruskal-Wallis Test	.319	Retain the null hypothesis.
2	The distribution of DC_INVnNUT is the same across categories of INVnNUT_String.	Independent-Samples Kruskal-Wallis Test	.001	Reject the null hypothesis.
3	The distribution of EC_INVnNUT is the same across categories of INVnNUT_String.	Independent-Samples Kruskal-Wallis Test	.000	Reject the null hypothesis.

Asymptotic significances are displayed. The significance level is .05.

```
*Nonparametric Tests: Independent Samples.
NPTESTS
  /INDEPENDENT TEST (BC_INVnEXP DC_INVnEXP EC_INVnEXP) GROUP (INVnEXP_St
g)
  /MISSING SCOPE=ANALYSIS USERMISSING=EXCLUDE
  /CRITERIA ALPHA=0.05  CILEVEL=95.
```

Nonparametric Tests

Hypothesis Test Summary

	Null Hypothesis	Test	Sig.	Decision
1	The distribution of BC_INVnEXP is the same across categories of INVnEXP_String.	Independent-Samples Kruskal-Wallis Test	.443	Retain the null hypothesis.
2	The distribution of DC_INVnEXP is the same across categories of INVnEXP_String.	Independent-Samples Kruskal-Wallis Test	.028	Reject the null hypothesis.
3	The distribution of EC_INVnEXP is the same across categories of INVnEXP_String.	Independent-Samples Kruskal-Wallis Test	.000	Reject the null hypothesis.

Asymptotic significances are displayed. The significance level is .05.

```
*Nonparametric Tests: Independent Samples.
NPTESTS
  /INDEPENDENT TEST (BC_INVnNA DC_INVnNA EC_INVnNA) GROUP (INVnNA_Strin
  /MISSING SCOPE=ANALYSIS USERMISSING=EXCLUDE
  /CRITERIA ALPHA=0.05  CILEVEL=95.
```

Nonparametric Tests

Hypothesis Test Summary

	Null Hypothesis	Test	Sig.	Decision
1	The distribution of BC_INVnNA is the same across categories of INVnNA_String.	Independent-Samples Kruskal-Wallis Test	.000	Reject the null hypothesis.
2	The distribution of DC_INVnNA is the same across categories of INVnNA_String.	Independent-Samples Kruskal-Wallis Test	.000	Reject the null hypothesis.
3	The distribution of EC_INVnNA is the same across categories of INVnNA_String.	Independent-Samples Kruskal-Wallis Test	.000	Reject the null hypothesis.

Asymptotic significances are displayed. The significance level is .05.

```
*Nonparametric Tests: Independent Samples.
NPTESTS
  /INDEPENDENT TEST (BC_NUTnEXP DC_NUTnEXP EC_NUTnEXP) GROUP (NUTnEXP_St
g)
  /MISSING SCOPE=ANALYSIS USERMISSING=EXCLUDE
  /CRITERIA ALPHA=0.05  CILEVEL=95.
```

Nonparametric Tests

Hypothesis Test Summary

	Null Hypothesis	Test	Sig.	Decision
1	The distribution of BC_NUTnEXP is the same across categories of NUTnEXP_String.	Independent-Samples Kruskal-Wallis Test	.813	Retain the null hypothesis.
2	The distribution of DC_NUTnEXP is the same across categories of NUTnEXP_String.	Independent-Samples Kruskal-Wallis Test	.317	Retain the null hypothesis.
3	The distribution of EC_NUTnEXP is the same across categories of NUTnEXP_String.	Independent-Samples Kruskal-Wallis Test	.000	Reject the null hypothesis.

Asymptotic significances are displayed. The significance level is .05.

```
*Nonparametric Tests: Independent Samples.
NPTESTS
  /INDEPENDENT TEST (BC_NUTnNA DC_NUTnNA EC_NUTnNA) GROUP (NUTnNA_Strin
  /MISSING SCOPE=ANALYSIS USERMISSING=EXCLUDE
  /CRITERIA ALPHA=0.05  CILEVEL=95.
```

Nonparametric Tests

Hypothesis Test Summary

	Null Hypothesis	Test	Sig.	Decision
1	The distribution of BC_NUTnNA is the same across categories of NUTnNA_String.	Independent-Samples Kruskal-Wallis Test	.000	Reject the null hypothesis.
2	The distribution of DC_NUTnNA is the same across categories of NUTnNA_String.	Independent-Samples Kruskal-Wallis Test	.000	Reject the null hypothesis.
3	The distribution of EC_NUTnNA is the same across categories of NUTnNA_String.	Independent-Samples Kruskal-Wallis Test	.007	Reject the null hypothesis.

Asymptotic significances are displayed. The significance level is .05.

```
*Nonparametric Tests: Independent Samples.
NPTESTS
  /INDEPENDENT TEST (BC_EXPnNA DC_EXPnNA EC_EXPnNA) GROUP (EXPnNA_Strin
  /MISSING SCOPE=ANALYSIS USERMISSING=EXCLUDE
  /CRITERIA ALPHA=0.05  CILEVEL=95.
```

Nonparametric Tests

Hypothesis Test Summary

	Null Hypothesis	Test	Sig.	Decision
1	The distribution of BC_EXPnNA is the same across categories of EXPnNA_String.	Independent-Samples Kruskal-Wallis Test	.000	Reject the null hypothesis.
2	The distribution of DC_EXPnNA is the same across categories of EXPnNA_String.	Independent-Samples Kruskal-Wallis Test	.000	Reject the null hypothesis.
3	The distribution of EC_EXPnNA is the same across categories of EXPnNA_String.	Independent-Samples Kruskal-Wallis Test	.000	Reject the null hypothesis.

Asymptotic significances are displayed. The significance level is .05.
