# Supplementary material for: A side-effect free method for identifying cancer drug targets
Source: Sci Rep. 2018 Apr 27;8:6669. doi: 10.1038/s41598-018-25042-2 (PMC5923273; doi:10.1038/s41598-018-25042-2)
Supplement: Supplementary file 3 — Supplementary Data Statistics [file 41598_2018_25042_MOESM3_ESM.zip › 20180306 Centrality_WocWct Output1.pdf]

```
*Nonparametric Tests: Independent Samples.
NPTESTS
  /INDEPENDENT TEST (BC7_WocWct DC7_WocWct EC7_WocWct) GROUP (FR7_Strin
  /MISSING SCOPE=ANALYSIS USERMISSING=EXCLUDE
  /CRITERIA ALPHA=0.05  CILEVEL=95.
```

Nonparametric Tests

```
[DataSet2] F:\20180306 Cancer Interactome Statistics\20180306 Centralit
cWct.sav
```

Hypothesis Test Summary

	Null Hypothesis	Test	Sig.	Decision
1	The distribution of BC7_WocWct is the same across categories of FR7_String.	Independent-Samples Kruskal-Wallis Test	.376	Retain the null hypothesis.
2	The distribution of DC7_WocWct is the same across categories of FR7_String.	Independent-Samples Kruskal-Wallis Test	.086	Retain the null hypothesis.
3	The distribution of EC7_WocWct is the same across categories of FR7_String.	Independent-Samples Kruskal-Wallis Test	.140	Retain the null hypothesis.

Asymptotic significances are displayed. The significance level is .05.

```
*Nonparametric Tests: Independent Samples.
NPTESTS
  /INDEPENDENT TEST (BC6_WocWct DC6_WocWct EC6_WocWct) GROUP (FR6_Strin
  /MISSING SCOPE=ANALYSIS USERMISSING=EXCLUDE
  /CRITERIA ALPHA=0.05  CILEVEL=95.
```

Nonparametric Tests

Hypothesis Test Summary

	Null Hypothesis	Test	Sig.	Decision
1	The distribution of BC6_WocWct is the same across categories of FR6_String.	Independent-Samples Kruskal-Wallis Test	.000	Reject the null hypothesis.
2	The distribution of DC6_WocWct is the same across categories of FR6_String.	Independent-Samples Kruskal-Wallis Test	.000	Reject the null hypothesis.
3	The distribution of EC6_WocWct is the same across categories of FR6_String.	Independent-Samples Kruskal-Wallis Test	.000	Reject the null hypothesis.

Asymptotic significances are displayed. The significance level is .05.

```
*Nonparametric Tests: Independent Samples.
NPTESTS
  /INDEPENDENT TEST (BC5_WocWct DC5_WocWct EC5_WocWct) GROUP (FR5_Strin
  /MISSING SCOPE=ANALYSIS USERMISSING=EXCLUDE
  /CRITERIA ALPHA=0.05  CILEVEL=95.
```

Nonparametric Tests

Hypothesis Test Summary

	Null Hypothesis	Test	Sig.	Decision
1	The distribution of BC5_WocWct is the same across categories of FR5_String.	Independent-Samples Kruskal-Wallis Test	.451	Retain the null hypothesis.
2	The distribution of DC5_WocWct is the same across categories of FR5_String.	Independent-Samples Kruskal-Wallis Test	.303	Retain the null hypothesis.
3	The distribution of EC5_WocWct is the same across categories of FR5_String.	Independent-Samples Kruskal-Wallis Test	.802	Retain the null hypothesis.

Asymptotic significances are displayed. The significance level is .05.

```
*Nonparametric Tests: Independent Samples.
NPTESTS
  /INDEPENDENT TEST (BC4_WocWct DC4_WocWct EC4_WocWct) GROUP (FR4_Strin(
  /MISSING SCOPE=ANALYSIS USERMISSING=EXCLUDE
  /CRITERIA ALPHA=0.05  CILEVEL=95.
```

Nonparametric Tests

Hypothesis Test Summary

	Null Hypothesis	Test	Sig.	Decision
1	The distribution of BC4_WocWct is the same across categories of FR4_String.	Independent-Samples Kruskal-Wallis Test	.000	Reject the null hypothesis.
2	The distribution of DC4_WocWct is the same across categories of FR4_String.	Independent-Samples Kruskal-Wallis Test	.000	Reject the null hypothesis.
3	The distribution of EC4_WocWct is the same across categories of FR4_String.	Independent-Samples Kruskal-Wallis Test	.000	Reject the null hypothesis.

Asymptotic significances are displayed. The significance level is .05.

```
*Nonparametric Tests: Independent Samples.
NPTESTS
  /INDEPENDENT TEST (BC3_WocWct DC3_WocWct EC3_WocWct) GROUP (FR3_Strin
  /MISSING SCOPE=ANALYSIS USERMISSING=EXCLUDE
  /CRITERIA ALPHA=0.05  CILEVEL=95.
```

Nonparametric Tests

Hypothesis Test Summary

	Null Hypothesis	Test	Sig.	Decision
1	The distribution of BC3_WocWct is the same across categories of FR3_String.	Independent-Samples Kruskal-Wallis Test	.000	Reject the null hypothesis.
2	The distribution of DC3_WocWct is the same across categories of FR3_String.	Independent-Samples Kruskal-Wallis Test	.000	Reject the null hypothesis.
3	The distribution of EC3_WocWct is the same across categories of FR3_String.	Independent-Samples Kruskal-Wallis Test	.000	Reject the null hypothesis.

Asymptotic significances are displayed. The significance level is .05.

```
*Nonparametric Tests: Independent Samples.
NPTESTS
  /INDEPENDENT TEST (BC2_WocWct DC2_WocWct EC2_WocWct) GROUP (FR2_Strin
  /MISSING SCOPE=ANALYSIS USERMISSING=EXCLUDE
  /CRITERIA ALPHA=0.05  CILEVEL=95.
```

Nonparametric Tests

Hypothesis Test Summary

	Null Hypothesis	Test	Sig.	Decision
**1**	The distribution of BC2_WocWct is the same across categories of FR2_String.	Independent-Samples Mann-Whitney U Test	.000	Reject the null hypothesis.
**2**	The distribution of DC2_WocWct is the same across categories of FR2_String.	Independent-Samples Mann-Whitney U Test	.000	Reject the null hypothesis.
**3**	The distribution of EC2_WocWct is the same across categories of FR2_String.	Independent-Samples Mann-Whitney U Test	.000	Reject the null hypothesis.

Asymptotic significances are displayed. The significance level is .05.

```
*Nonparametric Tests: Independent Samples.
NPTESTS
  /INDEPENDENT TEST (BC1_WocWct DC1_WocWct EC1_WocWct) GROUP (FR1_Strin
  /MISSING SCOPE=ANALYSIS USERMISSING=EXCLUDE
  /CRITERIA ALPHA=0.05  CILEVEL=95.
```

Nonparametric Tests

Hypothesis Test Summary

	Null Hypothesis	Test	Sig.	Decision
**1**	The distribution of BC1_WocWct is the same across categories of FR1_String.	Independent-Samples Kruskal-Wallis Test	.022	Reject the null hypothesis.
**2**	The distribution of DC1_WocWct is the same across categories of FR1_String.	Independent-Samples Kruskal-Wallis Test	.000	Reject the null hypothesis.
**3**	The distribution of EC1_WocWct is the same across categories of FR1_String.	Independent-Samples Kruskal-Wallis Test	.001	Reject the null hypothesis.

Asymptotic significances are displayed. The significance level is .05.
